# CDK12 loss drives prostate cancer progression, transcription-replication conflicts, and synthetic lethality with paralog CDK13

**DOI:** 10.1016/j.xcrm.2024.101758

**Published:** 2024-10-04

**Authors:** Jean Ching-Yi Tien, Jie Luo, Yu Chang, Yuping Zhang, Yunhui Cheng, Xiaoju Wang, Jianzhang Yang, Rahul Mannan, Somnath Mahapatra, Palak Shah, Xiao-Ming Wang, Abigail J. Todd, Sanjana Eyunni, Caleb Cheng, Ryan J. Rebernick, Lanbo Xiao, Yi Bao, James Neiswender, Rachel Brough, Stephen J. Pettitt, Xuhong Cao, Stephanie J. Miner, Licheng Zhou, Yi-Mi Wu, Estefania Labanca, Yuzhuo Wang, Abhijit Parolia, Marcin Cieslik, Dan R. Robinson, Zhen Wang, Felix Y. Feng, Jonathan Chou, Christopher J. Lord, Ke Ding, Arul M. Chinnaiyan

**Affiliations:** 1 Michigan Center for Translational Pathology, University of Michigan, Ann Arbor, MI, USA; 2 Department of Pathology, University of Michigan, Ann Arbor, MI, USA; 3 State Key Laboratory of Chemical Biology, Shanghai Institute of Organic Chemistry, Chinese Academy of Sciences, Shanghai 200032, People's Republic of China; 4 International Cooperative Laboratory of Traditional Chinese Medicine Modernization and Innovative Drug Discovery of Chinese Ministry of Education (MOE), Guangzhou City Key Laboratory of Precision Chemical Drug Development, College of Pharmacy, Jinan University, Guangzhou 511400, People's Republic of China; 5 The CRUK Gene Function Laboratory and Breast Cancer Now Toby Robins Research Centre, The Institute of Cancer Research, SW3 6JB London, UK; 6 Department of Genitourinary Medical Oncology and David H. Koch Center for Applied Research of Genitourinary Cancer, University of Texas MD Anderson Cancer Center, Houston, TX, USA; 7 Vancouver Prostate Centre, Vancouver General Hospital and Department of Urologic Sciences, University of British Columbia, Vancouver, BC V6H 3Z6, Canada; 8 Rogel Cancer Center, University of Michigan, Ann Arbor, MI, USA; 9 Departments of Radiation Oncology and Urology, University of California, San Francisco, San Francisco, CA, USA; 10 Helen Diller Family Comprehensive Cancer Center, University of California, San Francisco, San Francisco, CA, USA; 11 Division of Hematology/Oncology, Department of Medicine, University of California, San Francisco, San Francisco, CA, USA; 12 Department of Urology, University of Michigan, Ann Arbor, MI, USA; 13 Howard Hughes Medical Institute, University of Michigan, Ann Arbor, MI, USA

**Keywords:** CDK12, CDK13, prostate cancer, *Cdk12* knockout, transcription-replication conflicts, R-loops, paralog-based synthetic lethality

## Abstract

Biallelic loss of cyclin-dependent kinase 12 (*CDK12*) defines a metastatic castration-resistant prostate cancer (mCRPC) subtype. It remains unclear, however, whether *CDK12* loss drives prostate cancer (PCa) development or uncovers pharmacologic vulnerabilities. Here, we show *Cdk12* ablation in murine prostate epithelium is sufficient to induce preneoplastic lesions with lymphocytic infiltration. In allograft-based CRISPR screening, *Cdk12* loss associates positively with *Trp53* inactivation but negatively with *Pten* inactivation. Moreover, concurrent *Cdk12*/*Trp53* ablation promotes proliferation of prostate-derived organoids, while *Cdk12* knockout in *Pten*-null mice abrogates prostate tumor growth. In syngeneic systems, *Cdk12*/*Trp53*-null allografts exhibit luminal morphology and immune checkpoint blockade sensitivity. Mechanistically, *Cdk12* inactivation mediates genomic instability by inducing transcription-replication conflicts. Strikingly, *CDK12*-mutant organoids and patient-derived xenografts are sensitive to inhibition or degradation of the paralog kinase, CDK13. We therein establish *CDK12* as a *bona fide* tumor suppressor, mechanistically define how *CDK12* inactivation causes genomic instability, and advance a therapeutic strategy for *CDK12-*mutant mCRPC.

## Highlights

• *Cdk12* ablation induces preneoplastic prostate lesions with T cell infiltration
• *Cdk12* loss mediates genomic instability through transcription-replication conflicts
• *Cdk12*/*Trp53* knockout in murine allografts sensitizes to immune checkpoint blockade
• *CDK12* loss sensitizes paralog-based synthetic lethality via targeting CDK13

## Graphical abstract

**Figure:**
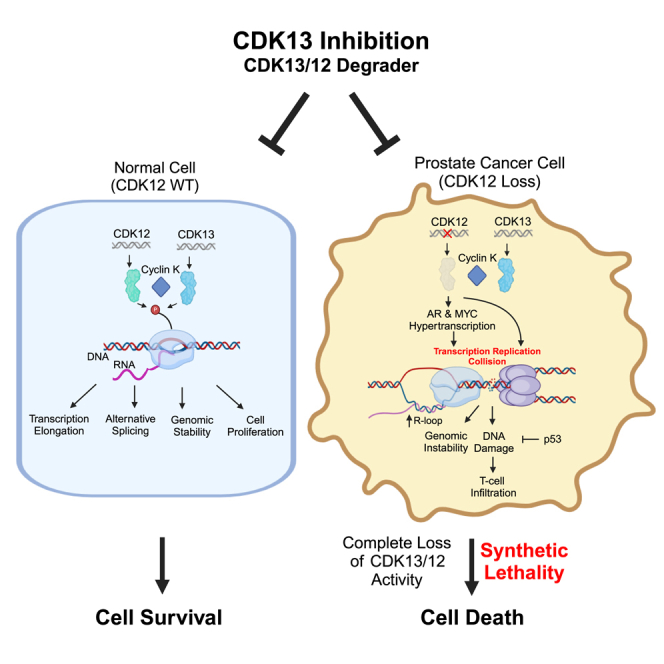

## Teaser

Tien et al. employ mouse models to define *Cdk12* as a bona fide prostate cancer tumor suppressor gene. *Cdk12* loss promotes transcription-replication conflicts, inducing DNA damage. Murine and human prostate tumors with inactive *CDK12* exhibit paralog-based synthetic lethality upon pharmacologic targeting of CDK13—a strategy with potential clinical application.

## Introduction

Cyclin-dependent kinases (CDKs) fall into two categories: cell cycle regulatory CDKs (e.g., CDK4 and 6), which drive cell cycle progression, and transcriptional CDKs (CDK7, 8, 9, 12, and 13), which regulate gene expression.^1^ CDK12 is a transcriptional CDK that associates with DNA in protein-coding and enhancer regions.^2^ Upon binding its cognate cyclin (cyclin K), CDK12 phosphorylates Ser2 residues within the C-terminal domain (CTD) of RNA polymerase II (Pol-II).^3^ This facilitates recruitment of phosphorylated-CTD-associated proteins required for transcriptional elongation^3^^,^^4^^,^^5^—a process thought to be important for expression of long genes containing numerous exons.^4^ CDK12 also regulates alternative splicing^6^^,^^7^ and pre-mRNA processing,^8^^,^^9^ while repressing intronic polyadenylation.^10^ Genetic and pharmacologic targeting has shown CDK12 to transcriptionally regulate DNA damage response (DDR) genes,^4^^,^^11^^,^^12^^,^^13^ while CDK12 loss of function reduces RAD51 focus formation and promotes *in vitro* poly (ADP-ribose) polymerase inhibitor (PARPi) sensitivity.^4^^,^^10^^,^^14^^,^^15^

CDK13 is a CDK12 paralog exhibiting 92% kinase domain homology and a similar three-dimensional structure.^1^ Like CDK12, CDK13 binds cyclin K to exert Pol-II CTD kinase activity.^16^ Comparison of gene expression profiles from HCT116 colon cancer cells subjected to *CDK12* or *CDK13* knockdown demonstrated 75% overlap in affected transcripts.^12^ Fan et al. applied CRISPR-Cas9 technology to generate MV4-11 leukemia cell lines in which CDK12, CDK13, or both could be inhibited via administration of ATP analog NM-PPI.^17^ This approach revealed that inhibition of both kinases, but not either individually, was sufficient to reduce CTD phosphorylation and proliferation while promoting cell death.^17^ In both systems, DDR transcripts were predominantly regulated by CDK12.^12^ As such, maintenance of genomic stability depends on CDK12, while cell proliferation and survival depend on redundant actions of CDK12 and CDK13.

*CDK12*-inactivating mutations occur in several malignancies.^18^^,^^19^^,^^20^ For instance, biallelic *CDK12* loss is observed in ∼4% of serous ovarian carcinoma, characterizing a disease subtype with recurrent focal tandem duplications (FTDs).^20^ Notably, these tumors are genetically distinct from those bearing inactivating mutations in the homologous recombination (HR) regulators *BRCA1* and *BRCA2*.^20^ Additionally, whole-exome sequencing of 360 metastatic castration-resistant prostate cancer (mCRPC) samples—from Stand Up to Cancer (SU2C),^21^ MI-ONCOSEQ,^22^ and Michigan Legacy Tissue Program (MLTP)^23^—revealed biallelic alterations in 25/360 cases (6.9%). By contrast, primary prostate cancer (PCa) sequences in The Cancer Genome Atlas (TCGA) revealed biallelic *CDK12* alterations in only 6/498 cases (1.2%)—a finding indicating enrichment of *CDK12* inactivation in metastatic disease.^18^ Indeed, biallelic *CDK12* loss constitutes a unique mCRPC subtype, genetically distinct from those driven by ETS gene fusions, *SPOP* mutations, homologous recombination deficiency (HRD), and mismatch repair deficiency.^21^^,^^22^^,^^23^^,^^24^^,^^25^^,^^26^^,^^27^ *CDK12*-mutant tumors are characterized by a genomic instability pattern like that described in ovarian cancer,^20^ in which recurrent gains secondary to FTDs yield putative neo-antigens.^18^ To this end, mCRPC tumors with *CDK12* loss exhibit T cell infiltration.^18^ Despite these associations, it remains unclear whether *CDK12* represents a *bona fide* tumor suppressor gene which, when inactivated, can drive tumorigenesis. Furthermore, it is unclear whether *CDK12* loss renders prostate tumors susceptible to paralog-based synthetic lethality.^28^

Here, we develop *in vivo* and *in vitro* systems to test the impact of *Cdk12* ablation—both independently and in the context of other canonical mCRPC-related mutations. We provide compelling evidence that *Cdk12* is a tumor suppressor gene and demonstrate its loss promotes androgen receptor (AR) and MYC-mediated hypertranscription, transcription-replication conflicts (TRCs), and resultant DNA damage. *Cdk12* loss enhances tumorigenesis and progression in the setting of *Trp53* loss; however, it inhibits growth of *Pten*-null tumors. We establish bigenic *Cdk12/Trp53* loss as a syngeneic model of PCa that exhibits an AR+ luminal phenotype and demonstrate lymphocytic infiltration and immune checkpoint blockade (ICB) sensitivity in this system. Finally, we leverage paralog-based synthetic lethality to demonstrate that murine and human tumor tissue lacking functional CDK12 is sensitive to CDK13 inhibition and degradation—a finding with future clinical applicability in *CDK12*-mutant cancers.

## Results

### Prostate-specific *Cdk12* ablation induces preneoplastic lesions and T cell infiltration

Biallelic loss-of-function mutations in *CDK12* occur in ∼7% of mCRPC^18^; however, whether *CDK12* inactivation promotes prostate tumorigenesis is unknown. We employed genetically engineered mice in which *Cdk12* exons 3 and 4 are flanked by *loxP* sites (*Cdk12*^*f/f*^ mice).^29^ Cre-mediated recombination of these loci excises the kinase domain to yield a nonfunctional, truncated protein and reduced mRNA levels.^11^ We crossed *Cdk12*^*f/f*^ mice into a probasin Cre (*Pb-Cre*) line.^30^ The resulting mixed genetic background animals (*Cdk12*^*pc−/−*^ mice) lack functional CDK12 in prostate epithelial cells (Figure S1A).

In the *Pb-Cre* model, roughly half of the luminal epithelial cells (LECs) and a smaller proportion of basal cells (BCs) have Cre activity. Cre is highly expressed in LECs of ventral, dorsal, and lateral prostate (VP, DP, and LP, respectively) but present in a minority of anterior prostate (AP) LECs.^30^ Our *Cdk12*^*pc−/−*^ mice, therefore, exhibited loss of CDK12 protein expression in 38%, 59%, 60%, and 70% of AP, VP, DP, and LP epithelial cells, respectively (Figure S1B). *In situ* hybridization (ISH) broadly corroborated immunohistochemistry (IHC) findings (Figure S1B).

Young *Cdk12*^*pc−/−*^ mice displayed normal prostate histology (Figure S1B); however, prostates of 30- and 52-week-old *Cdk12*^*pc−/−*^ mice exhibited patchy epithelial hyperplasia with loss of nuclear polarity and isonucleosis (Figures S1C and S1D). Histologically atypical tissue occupied ∼2% of cross-sectional area in the *Cdk12*^*pc−/−*^ AP, VP, and LP, while accounting for 10% in the DP. In contrast, similar tissue accounted for <1% of cross-sectional area in all lobes of wild-type (WT) littermates (Figure S1E).

**Figure 1:**
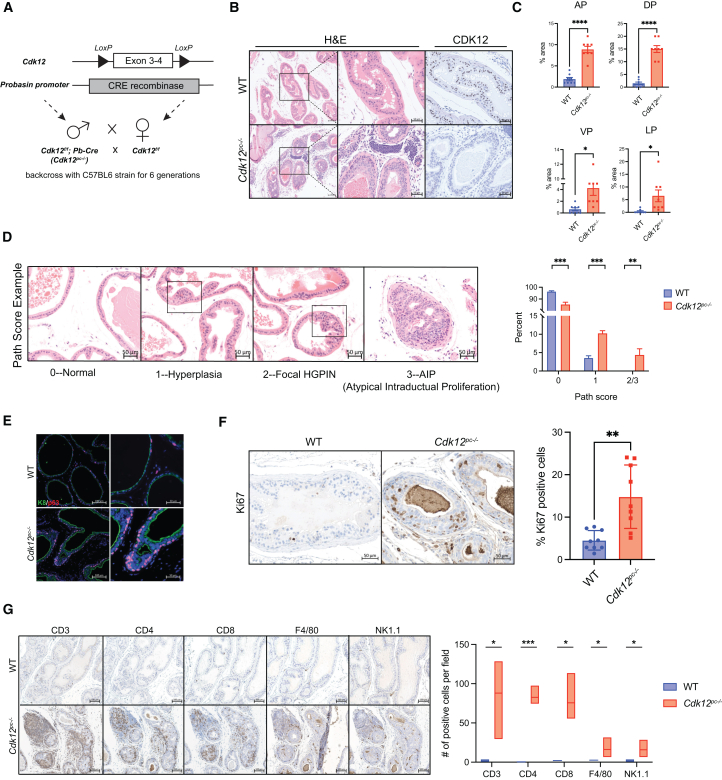
*Cdk12* ablation in the prostate epithelium induces neoplasia (A) Prostate epithelial *Cdk12* ablation scheme. (B) H&E staining and CDK12 immunohistochemistry in representative prostate samples from 52-week-old *Cdk12*^*pc−/−*^ mice (pure C57 background) and WT controls. Left panel scale bars, 100 μm. Other scale bars, 50 μm. (C) Bar graphs indicate percent cross-sectional area occupied by histologically abnormal tissue. AP, anterior prostate; DP, dorsal prostate; VP, ventral prostate; LP, lateral prostate. (*n* = 8/group). (D) Pathological scoring (Path score) of prostate from the same animals. Numerical scores assigned to normal tissue (0), hyperplasia (1), focal HGPIN (2), and AIP (3) (indicated by respective images). Scale bars, 50 μm. Bar graph shows percentage prostate cross-sectional area occupied by tissue of each path score (scores 2 and 3 added together). (*n* = 7–8/group). Statistical analysis with Mann-Whitney test. (E) Immunofluorescent staining of cytokeratin-8 (K8) and p63. 52-week-old *Cdk12*^*pc−/−*^ image shows an area of focal HGPIN with expansion of p63(+) BCs. Scale bars in left (100 μm) and right images (50 μm). (F) Ki67 immunohistochemistry in *Cdk12*^*pc−/−*^ or WT mice. Scale bars, 50 μm. Bar graph indicates percentage Ki67(+) cells per high-powered field (*n* = 9 images from 3 mice/group). Data represented as mean ± SEM. (G) Immunohistochemistry for immune cell markers, indicating T cell-predominant infiltrate surrounding lesions in *Cdk12*^*pc−/−*^ animals. Scale bars, 100 μm. Bar graph data indicate number of each cell type per high-powered field (*n* = 3–5 images from 3 mice/group). Box indicates standard deviation. ∗*p* < 0.05, ∗∗*p* < 0.01, ∗∗∗*p* < 0.001, ∗∗∗∗*p* < 0.0001. Student’s t test used in (C), (F), and (G). See also Figure S1.

To mitigate genetic variability, we backcrossed *Cdk12*^*pc−/−*^ animals with C57BL/6 mice for six generations to generate pure-background *Cdk12*-knockout mice (Figure 1A). Resulting animals displayed more marked prostate atypia, including areas of focal high-grade prostatic intraepithelial neoplasia (HGPIN) and atypical intraductal proliferation (AIP) (Figures 1B and 1C). Absent in WT prostate, these higher-grade lesions occupied 5% of *Cdk12*^*pc−/−*^ prostate cross-sectional area. Similarly, hyperplastic lesions occupied 12% of prostate cross-sections in *Cdk12*^*pc−/−*^ mice but <5% in WT controls (Figure 1D**)**. These preneoplastic lesions were characterized by [p63(+)] BC accumulation (Figure 1E**)** and increased cellular proliferation (Ki67 staining) (Figure 1F**)**. Like *CDK12*-mutant human mCRPC^188^, lesions in the *Cdk12*^*pc−/−*^ prostate exhibited T cell-predominant immune infiltration (Figure 1G**)**. In summary, *Cdk12* loss per se is sufficient to induce preneoplastic changes with corresponding immune infiltrates in the mouse prostate.

### *Cdk12*-null prostate-derived organoids are hyperplastic and display basal-luminal disorganization

To better define how *Cdk12* loss promoted tumorigenesis, we generated organoids from pure populations of *Cdk12*-null prostate epithelial cells. Given the mixture of CDK12(+) and CDK12(−) cells in the *Cdk12*^*pc−/−*^ prostate epithelium (Figure S1B), doing so required a system in which cells with *Cdk12* ablation could be identified and isolated. We, therefore, intercrossed *Cdk12*^*pc−/−*^ mice with *mT/mG* reporter mice. The latter harbor a constitutively expressed *td-Tomato/stop-floxed eGFP* construct. At baseline, *mT/mG* cells express *td-Tomato* [Tom(+)] and appear red. In the setting of Cre recombinase, the *td-Tomato* construct is excised and *eGFP* expressed [GFP(+)], such that cells appear green (Figure S2A). In *Pb-Cre*;*Cdk12*^*f/f*^;*mT/mG* mice, cells with active *Pb-Cre* (*Cdk12*-null) are GFP(+)/green.

**Figure 2:**
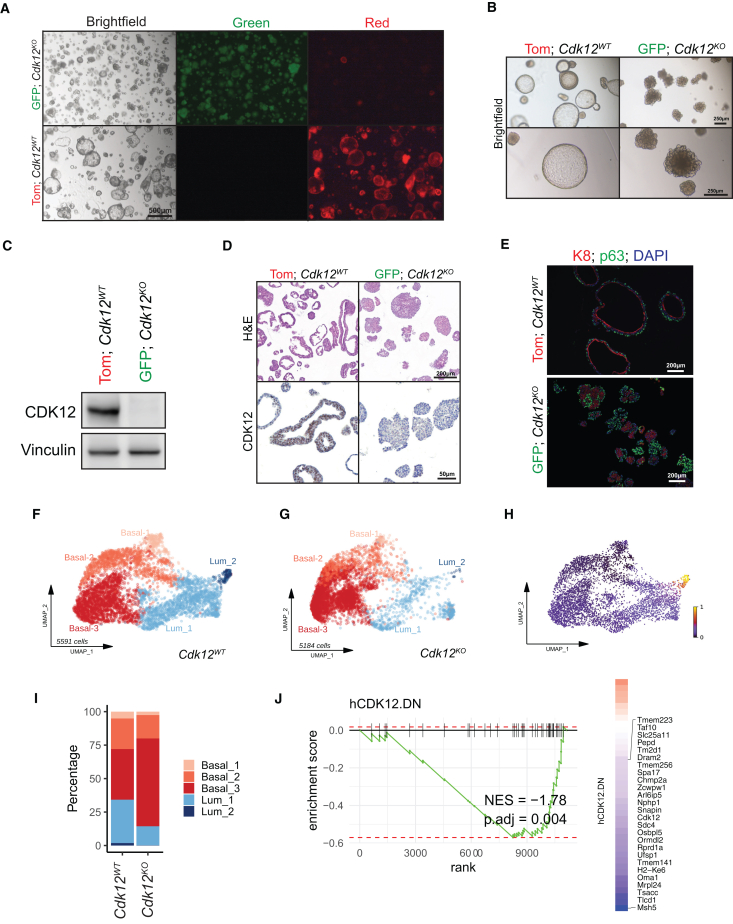
Organoids derived from the *Cdk12*^*pc−/−*^ prostate are morphologically abnormal, with impaired basal-luminal segregation (A and B) Images of organoids derived from *Pb-Cre*;*Cdk12*^*f/f*^;*mT/mG* prostate BCs (52-week time point). Tom indicates Td-tomato-expressing cells with wild-type *Cdk12* (*Cdk12*^*WT*^). GFP indicates GFP-expressing cells with *Cdk12* ablation (*Cdk12*^*KO*^). Scale bars, 500 μm in (A) and 250 μm in (B). (C) CDK12 immunoblot in *Cdk12*^*WT*^ vs. *Cdk12*^*KO*^ organoids. (Vinculin, loading control). (D) *Cdk12*^*KO*^ organoid morphology: H&E staining, and CDK12 immunohistochemistry. Scale bars 200 μm in top and 50 μm in bottom panels. (E) Immunofluorescence for cytokeratin-8 (K8) and p63 indicating basal-luminal disorganization in *Cdk12*^*KO*^ organoids. Scale bars, 200 μm. (F) Uniform Manifold Approximation and Projection (UMAP) of scRNA-seq from *Cdk12*^*WT*^ organoids (*n* = 3). The five identified cell states progress from Basal_1, Basal_2, Basal_3, Lum_1, to Lum_2. (G) UMAP of scRNA-seq from *Cdk12*^*KO*^ organoids (*n* = 3). (H) Cells from *Cdk12*^*KO*^ organoids (*n* = 3) projected into the UMAP of *Cdk12*^*WT*^. Pseudocolor indicates presence (yellow) or absence (purple) of *Cdk12* transcript. (I) Distributions of different cell states in *Cdk12*^*WT*^ and *Cdk12*^*KO*^ organoids. The most differentiated (Lum_2) population is lost in *Cdk12*^*KO*^ organoids. (J) GSEA for human *CDK12*-loss signature—shared down-regulated genes from human PCa with *CDK12* inactivation and siCDK12 knockdown LNCaP cells (18)— in *Cdk12*^*KO*^ organoids. Heatmap of logFC (*Cdk12*^*KO*^ vs. *Cdk12*^*WT*^ organoids) for genes in signature. Genes contributing to negative enrichment (leading edge) are labeled. See also Figure S2.

We used flow cytometry to isolate GFP(+) and Tom(+) BC and LEC from prostates of 52-week-old *Pb-Cre*;*Cdk12*^*f/f*^;*mT/mG* mice (Figure S2B) and observed the expected reduction of *Cdk12* transcript in GFP(+) cells (Figure S2C). GFP(+) and Tom(+) BCs respectively gave rise to organoids that were uniformly green and red in color (Figure 2A). Since LEC-derived organoids lacked this strict color segregation (Figure S2B), we employed only BC-derived organoids for all subsequent experiments. Upon initial analysis, red *Cdk12*^*WT*^ organoids exhibited normal murine prostate morphology—characterized by an organized epithelial layer surrounding a large lumen^31^; however, green *Cdk12*^*KO*^ organoids were smaller in size and lacked lumens (Figures 2A and 2B).

We applied multiple experimental systems to confirm this phenotype resulted specifically from *Cdk12* loss. First, we compared GFP(+) BC-derived organoids from *Pb-Cre*;*Cdk12*^*f/f*^;*mT/mG* and *Pb-Cre*;*Cdk12*^*+/+*^;*mT/mG* mice. Organoids derived from the former (*Cdk12*-null) were small and lacked lumens, whereas those derived from the latter (*Cdk12*-intact) had normal morphology (Figure S2D). Next, we isolated BCs from *Cdk12*^*f/f*^;*mT/mG* mice and treated *in vitro* with either Cre-expressing adenovirus or control adenovirus before generating organoids. Only organoids from Cre-expressing adenovirus-treated (*Cdk12*-null) cells demonstrated size reduction and absent lumens (Figure S2E).

We conducted further experiments comparing red *Cdk12*^*WT*^ and green *Cdk12*^*KO*^ organoids (Figure 2A**)**. In *Cdk12*^*KO*^ organoids, we confirmed loss of CDK12 protein with immunoblot (Figure 2C**)** and immunohistochemistry (Figure 2D**)**. Detailed observation of their morphology revealed *Cdk12*^*KO*^ organoids to be hyperplastic with disorganization of K8(+) LECs and p63(+) BCs (Figure 2E**)**. Single-cell RNA sequencing (scRNA-seq) (with velocity analysis) confirmed this, demonstrating reduced BC to LEC differentiation with resultant BC accumulation in *Cdk12*^*KO*^ samples (Figures 2F–2I). Gene set enrichment analysis based on pseudo-bulk profiles of organoid-derived LECs showed striking similarities between transcripts enriched in *Cdk12*^*KO*^ organoids and human PCa with biallelic *CDK12* loss^18^ (Figure 2J**)**. In all, *Cdk12*^*KO*^ organoids exhibit an abnormal phenotype consistent both with the preneoplastic lesions seen in prostates of *Cdk12*^*pc−/−*^ mice and with *CDK12*-mutant human PCa.

### *In vivo* CRISPR screen demonstrates *Cdk12* loss is positively associated with p53 inactivation

**Figure 3:**
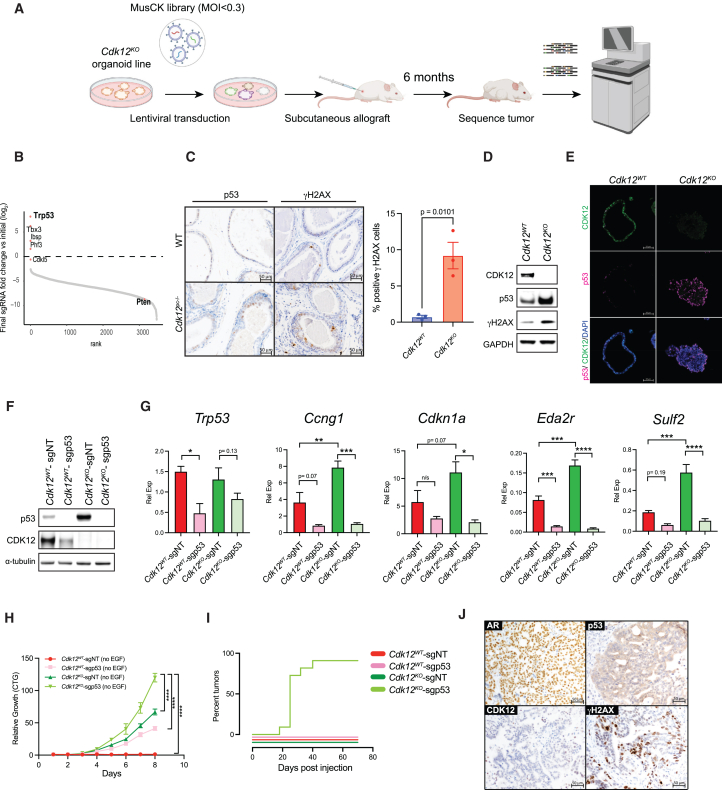
*Cdk12* and *Trp53* inactivating alterations interact to promote PCa (A) Workflow for CRISPR library screening of *Cdk12*-interacting genes. (B) Snake plot representing log_2_ fold change of guide RNAs in sequenced tumor samples described in (A). (*n* = 3/group in 2 unique experiments). (C) Immunohistochemistry for p53 (left panels) and γH2AX (right panels) in prostates of one-year-old WT and *Cdk12*^*pc−/−*^ mice. Scale bars, 50 μm. Bar graph indicates percent γH2AX(+) cells from average of 3–5 sections from each of 3 mice. Data represented as mean ± SEM. t test used for individual comparisons. (D) Protein expression of p53 and γH2AX in *Cdk12*^*WT*^ and *Cdk12*^*KO*^ organoids (GAPDH, loading control). (E) CDK12-p53 co-staining in *Cdk12*^*WT*^ and *Cdk12*^*KO*^ organoids. Scale bars, 50 μm. (F) CRISPR-mediated *Trp53* ablation in *Cdk12*^*WT*^ and *Cdk12*^*KO*^ organoids. sgp53 indicates *Trp53*-specific guide RNA. sgNT indicates control non-targeting guide RNA. (α-tubulin, loading control). (G) Relative expression (Rel Exp) levels of *Trp53* and p53 target genes in samples described in (F). (*n* = 3/group). Data represented as mean ± SEM. One-way AVOVA used for statistical comparisons. (H) Cell proliferation in organoids from groups indicated in (F) measured by CellTiter-Glo (CTG assay). (*n* = 3–4/group). Data represented as mean ± SEM. Two-way ANOVA used for statistical comparisons. (I) Kaplan-Meier plots indicating tumor formation during 70 days post-implantation of *Cdk12*^*WT*^ and *Cdk12*^*KO*^ organoids with or without *Trp53* ablation (*n* = 10/group). (J) Immunohistochemistry of AR, p53, CDK12, and γH2AX in *Cdk12*^*KO*^-sgp53 allografts. Scale bars, 50 μm. ∗*p* < 0.05; ∗∗*p* < 0.01; ∗∗∗*p* < 0.001; ∗∗∗∗*p* < 0.0001. See also Figure S3 and Table S1.

Clinically, *CDK12* inactivation displays variable overlap with other cardinal PCa mutations,^18^ suggesting its impact on tumorigenesis depends on mutational context. To identify mutations that positively and negatively interact with *Cdk12* loss, we applied CRISPR screening in our organoid model (Figure 3A). *Trp53*, a gene commonly inactivated in *CDK12*-mutant human tumors,^18^ emerged as the most significantly depleted gene in the screen (Figure 3B and Table S1).

In prostates of *Cdk12*^*pc−/−*^ mice, we observed both increased DNA damage (as indicated by γH2AX immunohistochemistry) and consequent p53 protein induction within preneoplastic lesions (Figure 3C**)**. In agreement, p53 signaling was among the most significantly upregulated pathways on scRNA-seq analysis of LECs isolated from the *Cdk12*-null prostate (Figures S3A–S3E). *Cdk12*^*KO*^ organoids phenocopied the *in vivo* findings, exhibiting elevated abundance of p53 and γH2AX protein (Figures 3D and 3E). In summary, *Cdk12* loss induces DNA damage and p53 expression, while cells with concurrent *Trp53* loss are preferentially enriched in allografts derived from *Cdk12*^*KO*^ organoids.

### Concomitant *Trp53* loss enhances tumorigenic potential of *Cdk12*-null prostate epithelial cells

We hypothesized p53 induction in *Cdk12*-null prostate epithelial cells represented a response to increased DNA damage and, hence, that these cells would display enhanced tumorigenic potential in the setting of *Trp53* loss. We tested this hypothesis by using CRISPR-Cas9 to ablate *Trp53* in *Cdk12*^*WT*^ and *Cdk12*^*KO*^ organoids. Both p53 protein levels (Figure 3F**)** and target gene expression (Figure 3G**)** were higher in *Cdk12*^*KO*^ organoids than in *Cdk12*^*WT*^ organoids when each was transfected with control single guide RNA (sgRNA) (*Cdk12*^*KO*^-sgNT and *Cdk12*^*WT*^-sgNT, respectively). Transfection of sgp53—to generate *Cdk12*^*WT*^-sgp53 and *Cdk12*^*KO*^-sgp53 organoids—yielded the appropriate reductions in p53 protein and target gene expression (Figures 3F and 3G). Organoids lacking either *Trp53* or *Cdk12* alone (*Cdk12*^*WT*^-sgp53 and *Cdk12*^*KO*^-sgNT, respectively) proliferated more rapidly than pure WT (*Cdk12*^*WT*^-sgNT) organoids. Strikingly, organoids lacking both genes (*Cdk12*^*KO*^-sgp53) proliferated more rapidly than either single-knockout organoid type (Figure 3H**)**.

We then implanted organoids into immunocompromised mice as subcutaneous allografts. While WT-sgNT, WT-sgp53, and *Cdk12*^*KO*^-sgNT organoids failed to form tumors during a 70-day monitoring period, 100% of *Cdk12*^*KO*^-sgp53 organoids generated tumors within 50 days of implantation (Figure 3I**)**. Immunohistochemistry for AR, p53, and γH2AX showed these tumors to be of prostate origin (AR(+)) and to display DNA damage (γH2AX(+)) (Figures 3J and S4A). Furthermore, tumors could be serially passaged in mice, exhibiting improved growth with each passage (Figure S4B). As such, concomitant loss of *Cdk12* and *Trp53* drives tumorigenesis beyond the loss of either factor alone. These findings mirror clinical data that demonstrate frequent association between inactivating mutations in *CDK12* and *TP53* in mCRPC.^18^

### *Cdk12*/*Trp53* double knockout allografts exhibit lymphocytic immune responses and increased sensitivity to ICB therapy

**Figure 4:**
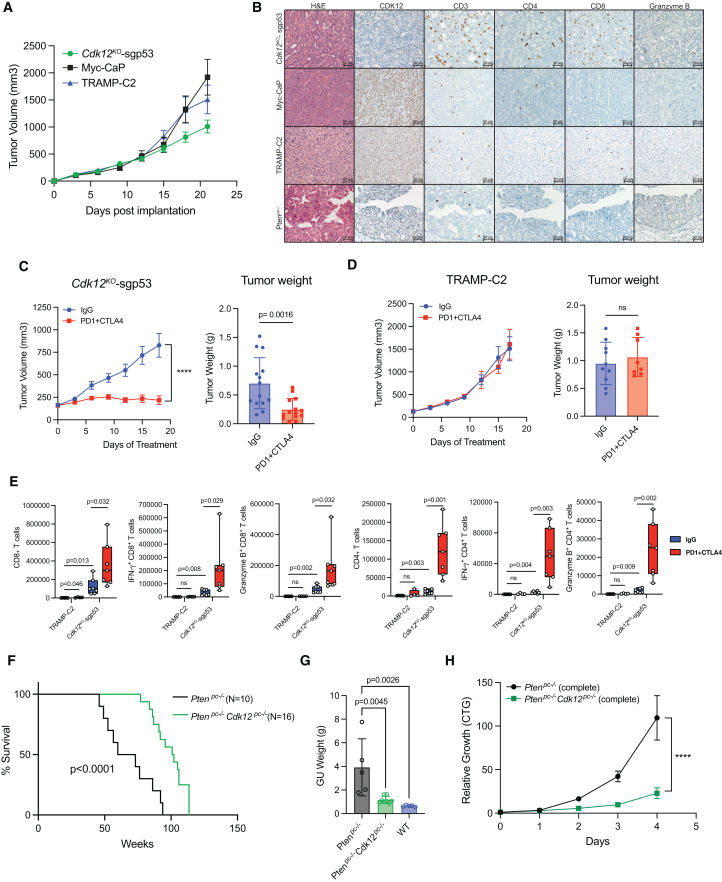
*Cdk12/Trp53* double knockout allografts exhibit lymphocytic immune responses and increased sensitivity to ICB therapy (A) Growth of *Cdk12*^*KO*^-sgp53, Myc-CaP, and TRAMP-C2 allografts in immunocompetent wild-type mice. (*n* = 10–15/group). (B) Immunohistochemistry of CDK12, T cell markers (CD3, CD4, CD8), and natural killer cell marker granzyme B in *Cdk12*^*KO*^-sgp53 allografts, Myc-CaP allografts, and TRAMP-C2 allografts, and prostates of *Pten*^*pc−/−*^ PCa mouse model. Scale bars, 50 μm. (C and D) Tumor growth curve and endpoint weights of *Cdk12*^*KO*^-sgp53 (C) and TRAMP-C2 (D) allografts treated with anti-PD1/CTLA4 cocktail. (*n* = 8–14/group). (E) Flow cytometry-based quantification of CD4(+) and CD8(+) T cells (total, IFNγ(+), granzyme B(+)) in *Cdk12*^*KO*^-sgp53 and TRAMP-C2 allograft samples +/− treatment with anti-PD1/CTLA4 cocktail. (*n* = 7–8/group). (F) Kaplan-Meier plots demonstrating survival of prostate-specific *Pten*^*pc−/−*^ and *Pten*^*pc−/−*^*Cdk12*^*pc−/−*^mice. (G) Genitourinary tract weights of *Pten*^*pc−/−*^ and *Pten*^*pc−/−*^*Cdk12*^*pc−/−*^ mice as well as wild-type mice (52 weeks). (H) Cell proliferation in complete media of epithelial cell organoids derived from *Pten*^*pc−/−*^ and *Pten*^*pc−/−*^*Cdk12*^*pc−/−*^ mice measured by CTG assay. (*n* = 4/group). Data represented as mean ± SEM. Data represented as mean ± SEM. One-way ANOVA for multiple comparisons (G), two-way ANOVA for multiple variables (C) and (E), and unpaired t test was used for tumor weight in (C) and (D). ∗∗∗∗*p* < 0.0001; ns, not significant. See also Figures S4 and S5.

To assess the immunogenicity of *Cdk12*^*KO*^-sgp53 organoid-derived tumors, we developed a syngeneic allograft line by implanting them subcutaneously in immunocompetent C57BL/6 mice. In this system, the tumors remained immunopositive for AR, K8, and p63, while demonstrating pronounced γH2AX staining (Figure S4C). Despite growing similarly to established PCa models (Figure 4A), *Cdk12*^*KO*^-sgp53 allografts elicited a T cell-predominant immune infiltrate not observed in Myc-CaP or TRAMP-C2 allografts, or prostate tumors of *Pten*-null mice (Figure 4B**)**. Most notably, CD8(+) T cells broadly permeated *Cdk12*^*KO*^-sgp53 allografts but were essentially absent in Myc-CaP, TRAMP-C2, and *Pten*^*pc−/−*^ samples.

Given these findings, we hypothesized *Cdk12*^*KO*^-sgp53 allografts would be sensitive to ICB. Indeed, equivalent doses of an anti-PD1/CTLA4 antibody cocktail strongly inhibited growth of these tumors (Figure 4C**)** but failed to curb that of TRAMP-C2 allografts (Figure 4D**)**. Strikingly, immune profiling of both tumor types revealed significantly more CD4(+) and CD8(+) T cells in *Cdk12*^*KO*^-sgp53 versus TRAMP-C2 samples (Figure 4E**)**. These differences were particularly pronounced with anti-PD1/CTLA4 therapy.

Interestingly, heightened immunogenicity in *Cdk12*^*KO*^-sgp53 allografts occurred despite the fact that FTDs—implicated in neo-antigen formation in *CDK12*-mutant PCA^18^—were not readily apparent on genomic sequencing (Figures S4D and S4E). Notably, however, LECs of the *Cdk12*-null prostate displayed upregulation of immunogenic pathways, suggesting other potential mechanisms underlying observed lymphocytic infiltration (Figures S3D and S3E). In summary, *Cdk12* loss induces proinflammatory cytokine expression in prostate epithelial cells that corresponds with T cell-predominant immune infiltrate like that seen in human tumors.^18^

### *Cdk12* loss mitigates progression of tumors with *Pten* inactivation

In contrast to the observed enrichment of *Trp53* sgRNA, our CRISPR screen revealed depletion of sgRNA targeting *Pten*, a gene rarely mutated in *CDK12*-mutant mCRPC (Figure 3B**)**. Given the mutual exclusivity of *CDK12* and *PTEN* inactivation in human tumors, we hypothesized loss of *Cdk12*, an activator of mammalian target of rapamycin (mTOR) signaling,^32^ would mitigate progression of tumors driven by *Pten* inactivation. To test the hypothesis, we intercrossed our *Cdk12*^*pc−/−*^ mice with animals from the *Pten*^*f/f*^ line. Strikingly, double knockout (*Pten*^*pc−/−*^*Cdk12*^*pc−/−*^) mice survived for a significantly longer time than mice with prostate-specific *Pten* ablation alone (*Pten*^*pc−/−*^ mice) (Figure 4F**)**. Genitourinary tract weight was also significantly greater in *Pten*^*pc−/−*^ mice versus *Pten*^*pc−/−*^*Cdk12*^*pc−/−*^ mice at 52 weeks of age (Figure 4G**)**, corresponding with more aggressive tumors on gross observation (Figure S5A). Histologically, prostates of *Pten*^*pc−/−*^ mice demonstrated aggressive adenocarcinoma, whereas those of *Pten*^*pc−/−*^*Cdk12*^*pc−/−*^ mice showed maintenance of normal ductal morphology and markedly reduced stromal infiltrate (Figure S5B). Similar findings were apparent in younger animals, as weights of individual prostate lobes were lower in *Pten*^*pc−/−*^*Cdk12*^*pc−/−*^ versus *Pten*^*pc−/−*^ mice at 24 weeks of age (Figure S5C).

We next aimed to determine whether the protective effect of *Cdk12* ablation in PCa driven by *Pten* loss could be recapitulated *in vitro*. To do so, we generated prostate epithelial organoids from 24-week-old *Pten*^*pc−/−*^ and *Pten*^*pc−/−*^*Cdk12*^*pc−/−*^ mice, observing growth of the latter to be significantly blunted (Figure 4H**)**. Histologically, *Pten*^*pc−/−*^*Cdk12*^*pc−/−*^ organoids displayed the absent-lumen phenotype but also demonstrated reduced cell proliferation (Ki67 staining) in the setting of equivalent Akt phosphorylation (Figure S5D). Consistent with the established positive regulation of mTOR signaling by CDK12,^32^ phosphorylated S6 was markedly reduced in *Pten*^*pc−/−*^*Cdk12*^*pc−/−*^ versus *Pten*^*pc−/−*^ organoids (Figure S5E). We confirmed these findings by using CRISPR-Cas9 to ablate *Pten* in the BC-derived *Cdk12*^*WT*^ and *Cdk12*^*KO*^ organoid lines described earlier. In agreement, *Cdk12*^*KO*^-sgPten organoids grew more slowly than *Cdk12*^*WT*^-sgPten organoids (Figure S5F and S5G). *Cdk12* ablation therein impairs growth of PCa driven by *Pten* loss. This aligns with the near-mutual exclusivity between *CDK12* and *PTEN* inactivating mutations in human mCRPC.^33^

### *Cdk12* loss induces hypertranscription, TRCs, R-loop formation, and consequent DNA damage

*Cdk12*-null cells are prone to DNA damage—both *in vivo* and in organoid models. In PCa, hypertranscription—mediated by trophic pathways including AR and Myc signaling—induces DNA damage.^34^^,^^35^ As a key mediator of transcriptional elongation, CDK12 itself may also mitigate collisions between DNA replisome and transcription machinery, an established cause of double-strand DNA breaks downstream of aberrant R-loop resolution^36^

**Figure 5:**
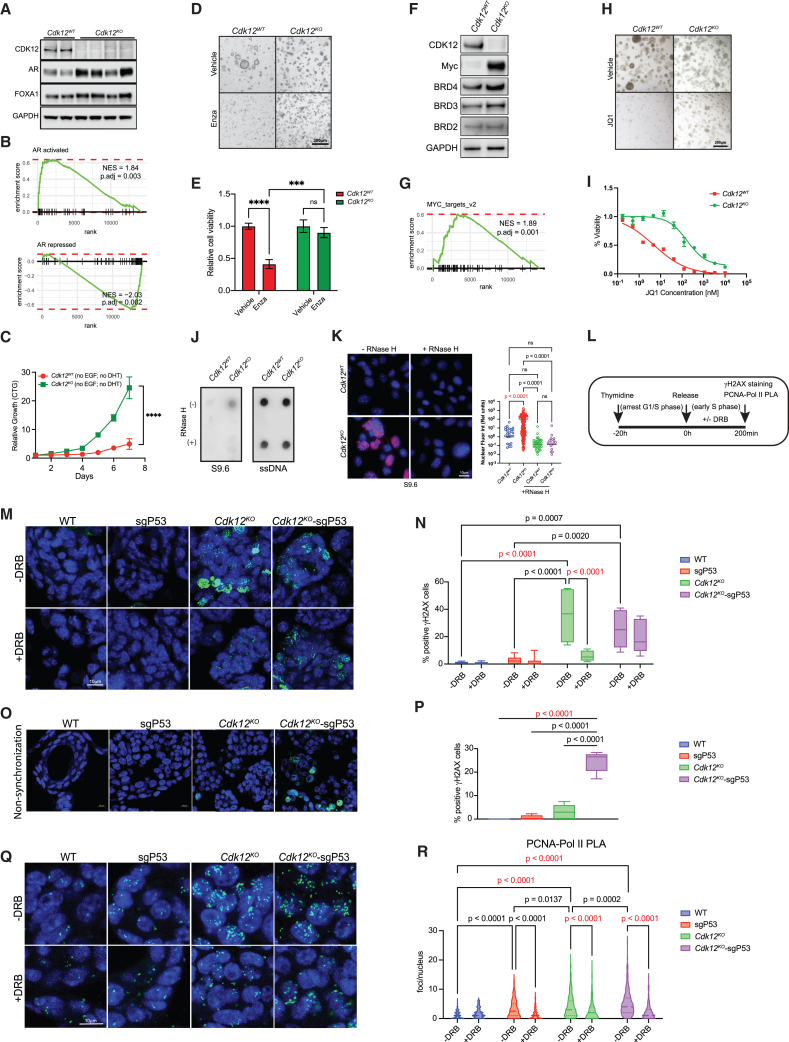
*Cdk12* ablation increases AR- and MYC-mediated signaling and promotes TRCs (A) Protein expression of CDK12, AR, and FOXA1 in multiple monoclonal *Cdk12*^*WT*^ and *Cdk12*^*KO*^ organoid lines. (GAPDH, loading control). (B) Gene set enrichment of AR target genes (activated and repressed) in *Cdk12*^*KO*^ organoids compared to *Cdk12*^*WT*^. (C) Proliferation of *Cdk12*^*WT*^ and *Cdk12*^*KO*^ organoids grown in the absence of epidermal growth factor (EGF) and dihydrotestosterone (DHT) as measured by the CTG assay. (*n* = 3 replicates per group in 2 unique experiments). (D and E) Morphology and viability quantification of *Cdk12*^*WT*^ and *Cdk12*^*KO*^ organoids subjected to enzalutamide (Enza) treatment. (*n* = 3 replicates per group in 2 unique experiments). ∗∗∗*p* < 0.001; ∗∗∗∗*p* < 0.0001; ns, not significant. (F) Protein expression of CDK12, MYC, BRD4, BRD3, and BRD2 in *Cdk12*^*WT*^ and *Cdk12*^*KO*^ organoid lines. (G) Gene set enrichment of MYC target genes in *Cdk12*^*KO*^ organoids compared to *Cdk12*^*WT*^. (H) Morphology of *Cdk12*^*WT*^ and *Cdk12*^*KO*^ organoid lines treated with JQ1 (1 μM). (*n* = 3/group in 2 unique experiments). (I) Viability curves and IC_50_ values for JQ1-treated *Cdk12*^*WT*^ and *Cdk12*^*KO*^ organoid lines. (J) Dot blot analysis quantifying R-loops in *Cdk12*^*WT*^ and *Cdk12*^*KO*^ organoids. RNase H1 treatment serves as a negative control. (K) Immunofluorescence images of R-loop (red) staining of *Cdk12*^*WT*^ and *Cdk12*^*KO*^ organoids (left) and quantification of fluorescence intensity (right). 100–200 cells/group. (L) Experimental workflow for identification of TRCs. Briefly, 2.5 mM of Thymidine was used to synchronize the cells, and 75 μM of DRB was used to inhibit transcription. (M) Representative immunofluorescence images of γH2AX staining in organoids treated as described in (L). (N) Quantification of γH2AX-positive cells in (M); (*n* = 6/group, 3 unique experiments conducted). (O) Representative immunofluorescence images of γH2AX staining in unsynchronized organoids. (P) Quantification of γH2AX-positive cells in (O); *n* = 6–8 per group (3 unique experiments conducted). (Q) Detection of TRC by PLA assay. (R) Quantification of PLA foci per nucleus in (Q); 100–400 cells analyzed per group (2 unique experiments conducted). Data represented as mean ± SEM. One-way ANOVA for multiple comparisons, two-way ANOVA for multiple variables.

Given the relationship between *CDK12* loss and castration resistance in human PCa,^37^^,^^38^ we first evaluated AR signaling in *Cdk12*^*KO*^ organoids. We found *Cdk12*^*KO*^ organoids to exhibit increased protein levels of AR and its coactivator FOXA1 (Figure 5A). In agreement, AR target gene expression signatures were enriched in the setting of *Cdk12* loss (Figure 5B**)**. The functional relevance of upregulated AR signaling in *Cdk12*^*KO*^ organoids was manifested in their ability to outgrow *Cdk12*^*WT*^ organoids in testosterone-depleted culture media (Figure 5C**)**. Furthermore, *Cdk12*^*KO*^ organoids were resistant to treatment with the anti-androgen enzalutamide (Figures 5D and 5E). *Cdk12* loss also induced upregulation of Myc protein, despite no significant changes in levels of the bromodomain proteins BRD2, BRD3, and BRD4 (Figure 5F**)**. In line with this, Myc target gene signatures were strongly induced in *Cdk12*^*KO*^ organoids (Figure 5G**)**. The higher Myc levels induced by the *Cdk12*-null state rendered these organoids resistant to the bromodomain inhibitor JQ1 (Figures 5H and 5I).

R-loops are DNA-RNA hybrid structures induced by hypertranscription that, without proper resolution, predispose DNA to double-strand breaks.^39^^,^^40^ We performed both dot blot assay and immunofluorescence staining using the S9.6 antibody (which binds DNA-RNA hybrids) and observed increased signal intensity in *Cdk12*^*KO*^ versus *Cdk12*^*WT*^ organoids (Figures 5J and 5K). DNA damage associated with unresolved R-loops in the setting of TRCs occurs, by definition, in S-phase. To determine the timing of CDK12 loss-mediated DNA damage, we subjected organoids to thymidine block, synchronizing cells in G1/S-phase. We then released cells from the block and, after 3.5 h (with cells still in early S-phase), stained for γH2AX (Figure 5L**)**. This approach revealed a marked increase in S-phase-specific γH2AX in *Cdk12*^*KO*^ versus *Cdk12*^*WT*^ organoids (Figure 5M and 5N). In the same experiment, sgp53 organoids displayed similar γH2AX staining to *Cdk12*^*WT*^ organoids, while *Cdk12*^*KO*^-sgp53 organoids exhibited similar staining to *Cdk12*^*KO*^ organoids. Treatment with RNA Pol-II inhibitor 5,6-dichloro-1-beta-D-ribofuranosylbenzimidazole (DRB) inhibited γH2AX accumulation in *Cdk12*^*KO*^ organoids, indicating that *Cdk12* loss induces DNA damage in a transcription-dependent manner. Interestingly, γH2AX staining in *Cdk12*^*KO*^-sgp53 organoids was present despite DRB treatment (Figures 5M and 5N). Since γH2AX was not induced by *p53* loss alone, we surmised that absence of *Cdk12* induces DNA damage, while concomitant absence of *p53* enables DNA damage to persist through multiple cell cycles. This model was supported by findings that, in asynchronized organoids, γH2AX staining was elevated over baseline only in the *Cdk12*^*KO*^-sgp53 group (Figures 5O and 5P).

To determine whether S-phase-specific DNA damage induced by *Cdk12* loss indeed corresponded with TRCs, we subjected organoids to an identical thymidine synchronization/release, followed by a proximity ligation assay (PLA) for proliferating cell nuclear antigen (PCNA, a DNA polymerase interacting protein) and RNA Pol-II (Figure 5L**)**. Labeled PLA foci indicate regions where DNA polymerase and RNA Pol-II are in close proximity. In this system, loss of *Cdk12* and *Trp53* independently induced PLA foci—though the increase was more pronounced in the *Cdk12*^*KO*^ group and the highest of all in the *Cdk12*^*KO*^-sgp53 group (Figures 5Q and 5R). In all groups, DRB treatment predictably reduced PLA focus number (Figures 5Q and 5R). As previously observed with CDK12 knockdown or pharmacologic inhibition,^4^^,^^41^ multiple DDR genes declined in *Cdk12*^*KO*^ organoids (Figure S5H). Some of these expression differences, however, depended on increased p53 levels in the *Cdk12* null state—as indicated by the fact that their expression returned toward WT levels in *Cdk12*^*KO*^-sgp53 organoids.

Together, these data reveal TRCs underpin *Cdk12* loss-induced DNA damage. These events are exacerbated in the setting of (AR and Myc-mediated) hypertranscription, while resultant double-strand DNA breaks persist if p53 function is simultaneously lost. In all, the model provides a mechanism for γH2AX increase in the *Cdk12*^*pc−/−*^ prostate and for the synergistic effect of combined *Cdk12/Trp53* loss in tumorigenesis.

### *CDK12* loss renders prostate epithelial tumor cells sensitive to CDK13 paralog inhibition

**Figure 6:**
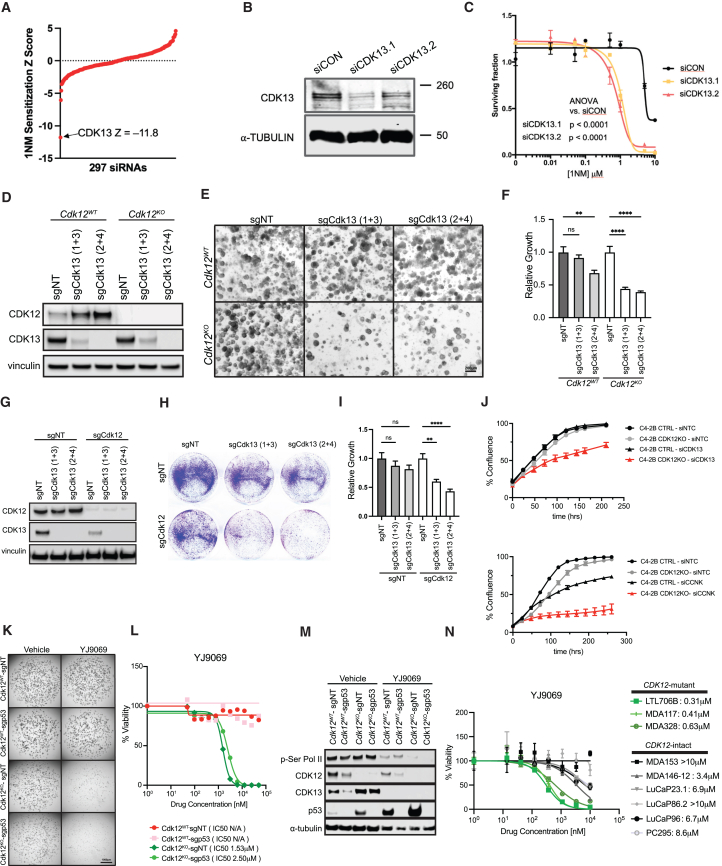
*Cdk12*^*KO*^ organoids and *CDK12*-mutant tumors are preferentially sensitive to a CDK13/12 degrader (A) Snake plot representing data from siRNA screen for CDK12 synthetic lethal effects via 1NM sensitivity in CDK12^as^ cells. Negative *Z* scores indicate CDK12 synthetic lethal effects, with CDK13 representing most profound effect. (B) Immunoblot indicating CDK13 gene silencing with two different siRNAs (siCDK13.1 and siCDK13.2). (C) Curve depicting cell survival in 1NM-exposed CDK12^as^ cells transfected with one of two unique CDK13 siRNAs (siCDK13.1 and siCDK13.2) or control siRNA (siCON). (D) CRISPR-mediated *Cdk13* (sgCdk13(1 + 3), or sgCdk13(2 + 4)) knockout in *Cdk12*^*WT*^ and *Cdk12*^*KO*^ organoids harvested on day 5 after lentiviral transduction. Protein expression of CDK12 and CDK13 in organoids (Vinculin, loading control). (E) Bright-field images of organoids described in (D). Scale bars, 200 μm. (F) Relative growth quantification from images in (E). (*n* = 3/group). (G) CRISPR ablation of *Cdk12* (sgCdk12) and *Cdk13* (sgCdk13(1 + 3), or sgCdk13(2 + 4)) in Myc-CaP cells. Protein expression of CDK12 and CDK13 in Myc-CaP cells treated with indicated sgRNAs. (H) Colony formation assay showing survival in cells treated with indicated sgRNAs (representative data from 3 unique experiments). (I) Relative growth quantification from images in (H) (analysis of 11 high-powered fields per sample over 2 unique experiments). (J) (Top panel) C4-2B cells subjected to CRISPR-based *CDK12* ablation (*CDK12*KO) or control sgRNA (C4-2B CTRL): percent confluence with siRNA-based *CDK13* knockdown (si*CDK13*) or control siRNA (siNTC). (Bottom panel) C4-2B *CDK12*KO and C4-2B CTRL cells: percent confluence with siRNA-based *CCNK* knockdown or control siRNA treatment. (*n* = 3/group). (K) Images of *Cdk12*^*WT*^ and *Cdk12*^*KO*^ organoids (with or without *Trp53* ablation) following treatment with CDK12/13 degrader (YJ9069). sgp53 indicates *Trp53* ablation, while sgNT indicates intact *Trp53*. Scale bars, 1,000 μm. (L) Viability curves and IC_50_ values for YJ9069 treatment of groups described in (K). (*n* = 4) (M) Protein expression of p-Ser RNA Pol-II, CDK12, CDK13, and p53 in *Cdk12*^*WT*^ and *Cdk12*^*KO*^ organoids with or without *Trp53* ablation subjected to YJ9069 degrader or vehicle treatment. (N) IC_50_ of organoids derived from PDX lines with WT *CDK12* (MDA153, MDA146-12, LuCaP23.1, LuCaP86.2, LuCaP96, PC295) and inactivating *CDK12* mutation (LTL706B, MDA117, MDA328). (*n* = 3 per line). Data represented as mean ± SEM. One-way ANOVA for multiple comparisons, two-way ANOVA for multiple variables, ∗∗*p* < 0.01, ∗∗∗∗*p* < 0.0001. See also Figures S6, S7 and Table S2.

To identify candidate synthetic lethal effects associated with CDK12 dysfunction, we used a previously described CRISPR-Cas9 engineered HeLa cell line, CDK12^as^ (“analogue sensitive”) cells, in which the only functional *CDK12* allele contains a kinase domain missense mutation rendering it sensitive to inhibition by the cell-permeable adenine analog 1-NM-PP1 (1NM)^42^ (Figure S6A). We validated CDK12^as^ cells, demonstrating that 1NM administration reduced Pol-II CTD phosphorylation, diminished nuclear RAD51 foci after ionizing radiation and induced PARPi sensitivity (Figures S6B–S6G). As expected, re-expression of WT CDK12 reversed PARPi sensitivity (Figures S6H and S6I). We reasoned genes dysregulated in *CDK12*-mutant cancers might be CDK12 synthetic lethal genes. We, therefore, screened CDK12^as^ cells with a small interfering RNA (siRNA) library targeting 297 candidate genes with putative functional relationships to CDK12 (Table S2; Figures S6J–S6L, see STAR Methods). The screen identified *CDK13* siRNA as most deleterious for survival of 1NM-treated CDK12^as^ cells (Figure 6A). We confirmed the ability of multiple *CDK13* siRNAs to induce cell death when CDK12 was inhibited by 1NM (Figures 6B and 6C).

Since CDK13 is a CDK12 paralog, we asked whether our *Cdk12*^*KO*^ organoids were susceptible to paralog-based synthetic lethality. Indeed, CRISPR-based ablation of *Cdk13* preferentially impaired organoid growth of *Cdk12*^*KO*^ versus *Cdk12*^*WT*^ organoids (Figures 6D–6F). We then developed an isogenic model of *Cdk12* loss by employing CRISPR-Cas9 to ablate *Cdk12* in Myc-CaP cells (Figure 6G**)**. While *Cdk12* loss reduced cell number on colony formation assay, cell survival was further inhibited with co-ablation of *Cdk12* and *Cdk13* (Figures 6H and 6I). We further employed CRISPR to ablate *CDK12* in the C4-2B PCa line. When subjected to siRNA-mediated *CDK13* knockdown, these cells exhibited greater growth reduction than si*CDK13*-treated C4-2B cells with intact *CDK12* (Figure 6J**)**. Knockdown of cyclin K (*CCNK*)—the obligate binding partner required for kinase activity of both CDK12 and CDK13—also preferentially inhibited proliferation of *CDK12-*knockout C4-2B cells (Figure 6J**)**. Taken together, these data substantiate CDK12 and 13 as synthetic lethal paralogs.^28^

We next tested the effectiveness of pharmacologically targeting CDK13 in *Cdk12*^*KO*^ organoids. Since selective CDK13 inhibitors/degraders are still in development, we employed the combined CDK13/12 degrader YJ9069 (Figure S7A) presuming that active CDK12/13 levels would be lower in treated *Cdk12*^*KO*^ versus *Cdk12*^*WT*^ organoids—therein providing a therapeutic window to exploit paralog redundancy.^43^ Treatment with YJ9069 markedly reduced the viability of *Cdk12*^*KO*^-sgNT and *Cdk12*^*KO*^-sgp53 organoids versus *Cdk12*^*WT*^-sgNT and *Cdk12*^*WT*^-sgp53 organoids (Figures 6K and 6L). Ser Pol-II phosphorylation declined in YJ9069-treated organoids with intact *Cdk12*; however, it was essentially absent in YJ9069-treated *Cdk12*^*KO*^ organoids (Figure 6M**)**. Similar to YJ9069, CDK13/12 inhibitors (YJ5118 and THZ531) exhibited more potent effects on cell viability in *Cdk12*^*KO*^ organoids compared to *Cdk12*^*WT*^ (Figure S7B). In addition, *CDK12*-knockout C4-2B cells showed preferential susceptibility to THZ531 treatment over C4-2B cells with intact *CDK12* (Figures S7C and S7D). Finally, we analyzed the efficacy of CDK13/12 degrader therapy in our *Cdk12*-null Myc-CaP cells. In this system, YJ9069 effectively inhibited growth of sgCdk12 Myc-CaP cells (half-maximal inhibitory concentration, IC_50_ 3.4 μM) but had no impact on Myc-CaP cells treated with control sgRNA (sgNT), even at a concentration of 20 μM (Figure S7E). These results underscore the effectiveness of CDK13-targeting paralog-based synthetic lethality in cells lacking *Cdk12*.

Next, we analyzed the impact of YJ9069 treatment on patient-derived xenograft (PDX) lines with biallelic *CDK12* inactivating mutations (Figure S7F). We successfully grew tumor chunks from new PDX line LTL706B in mouse renal capsules and then confirmed immunopositivity for PCa markers (AR, KRT8, and PSMA) and absence of CDK12 (Figure S7G). After propagating *in vivo*, we generated an LTL706B organoid line (Figure S7H). We also developed organoid models from (established *CDK12*-mutant PDXs) MDA117 and MDA328. *In vitro* treatment of each organoid line with YJ9069 revealed IC_50_ values lower by an order of magnitude than those of established *CDK12-*intact PDX lines (MDA153, MDA146-12, LuCaP23.1, LuCaP86.2, LuCap96, PC295) (Figures 6N and S7F).

**Figure 7:**
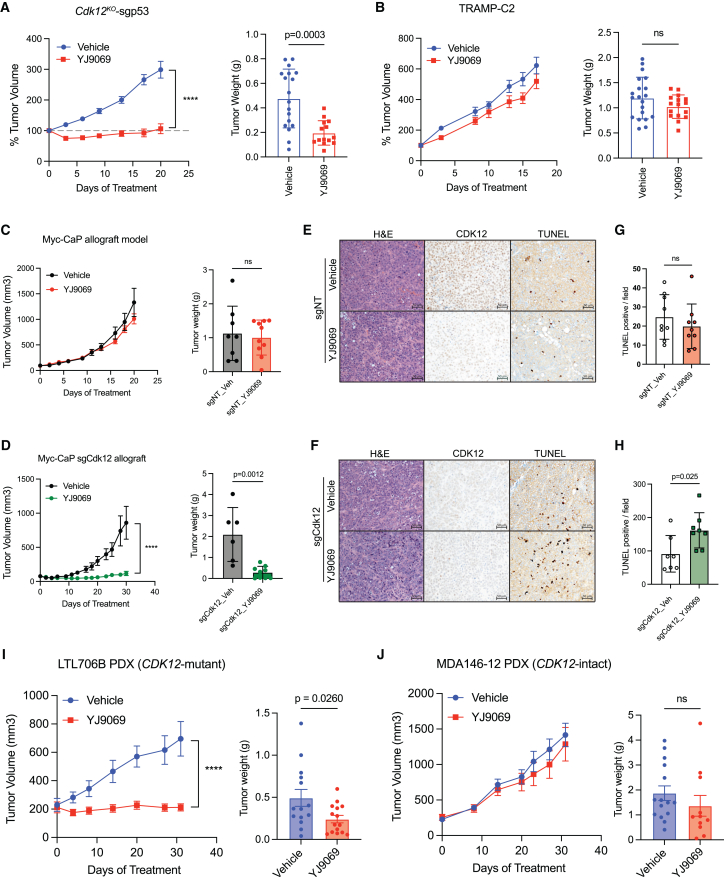
CDK13/12 degrader inhibits *CDK12*-mutant tumor growth *in vivo* (A) *In vivo* treatment of *Cdk12*^*KO*^-sgp53 allografts with YJ9069 or vehicle: line graph indicates tumor volume normalized to baseline. Bar graph indicates tumor weight at endpoint. (*n* = 9–10 mice, each with 2 tumors, per group) (B) *In vivo* treatment of TRAMP-C2 allografts with YJ9069 or vehicle: graphs as indicated in (A) (*n* = 9–10 mice, each with 2 tumors, per group). (C and D) Unmodified (sgNT-treated) Myc-CaP allografts (C) or sgCdk12-treated Myc-CaP allografts (D) subjected to *in vivo* YJ9069 treatment: line graphs indicate tumor volume. Bar graphs indicate tumor weight at end of treatment time course. (*n* = 6–9/group). (E–H) CDK12 immunohistochemistry and TUNEL staining of unmodified (sgNT-treated) Myc-CaP allografts (E) and sgCdk12-treated Myc-CaP allografts (F). Bar graphs (G and H) indicate quantification of TUNEL(+) cells per high-powered field (scale bars, 50 μm). (I and J) YJ9069 treatment of subcutaneously implanted PDX lines, LTL706B (*CDK12*-mutant), and MDA146-12 (intact *CDK12*). Graphs indicate tumor volume (*n* = 8–9 mice, each with 2 tumors per group). Two-way ANOVA used for tumor volume in (A), (D), and (I); unpaired t test used for tumor weight in (A–D) and (I and J) and TUNEL staining (G and H). ∗∗∗∗*p* < 0.0001; ns, not significant. See also Figure S7.

We then sought to determine whether CDK13 degradation was also effective *in vivo*. Indeed, intravenous administration of YJ9069 significantly blunted growth of subcutaneous *Cdk12*^*KO*^-sgp53 allografts but not allografts from the established TRAMP-C2 model (Figures 7A and 7B). In agreement, sgCdk12 Myc-CaP allografts demonstrated significantly reduced tumor growth versus sgNT Myc-CaP allografts when treated with YJ9069 (Figures 7C and 7D). YJ9069-treated sgCdk12 Myc-CaP tumors exhibited increased apoptosis (Figures 7E–7H).

Finally, we aimed to determine if *CDK12*-mutant PDX tumors shared the same *in vivo* sensitivity to CDK13/12 degrader therapy. Indeed, subcutaneous allografts derived from *CDK12*-mutant LTL706B were highly sensitive to YJ9069 therapy (Figure 7I), while those derived from *CDK12*-intact MDA146-12 showed similar growth with YJ9069 and vehicle treatment (Figure 7J**)**. In all, *CDK12* loss increases dependence on CDK13 to render PCa cells sensitive to CDK13/12 inhibitors and degraders. These findings suggest a vulnerability that may be leveraged clinically in *CDK12*-mutant cancers, ideally with CDK13-selective inhibitors/degraders.

## Discussion

Clinical and pre-clinical data amassed suggest *CDK12* has a tumor suppressor function in serous ovarian carcinoma^20^^,^^44^^,^^45^ and triple-negative breast cancer (TNBC).^46^ In PCa, our prior clinical sequencing studies established a relationship between biallelic *CDK12* inactivation and mCRPC.^18^ Nonetheless, whether *CDK12* loss per se could promote prostate tumorigenesis or progression remained unknown. We addressed that question here, finding conditional *Cdk12* ablation in mouse prostate epithelium is sufficient to induce preneoplastic lesions (including focal HGPIN and AIP). These data reveal a *bona fide* tumor suppressor role for *Cdk12* in PCa. Organoids derived from the *Cdk12*^*KO*^ prostate exhibit an abnormal compact morphology and basal-to-luminal differentiation defects like those seen with loss of other PCa tumor suppressor genes.^31^ They also exhibit gene expression signatures and enzalutamide resistance consistent with human *CDK12*-mutant PCa.^18^ Taken together, our *in vivo* and *ex vivo* systems define *Cdk12* as a tumor suppressor gene while reliably modeling aspects of human disease.

The tumor suppressor function of *CDK12* has been attributed to its regulation of DDR genes. For instance, in ovarian cancer, *CDK12* loss downregulates transcripts in the HR DNA repair pathway—a phenotype attributed to reduced CDK12/cyclin K-mediated transcriptional elongation of the corresponding genes.^47^ *CDK12* is also implicated as a positive regulator of *BRCA* expression in both ovarian^48^ and TNBC cells.^41^ Conversely, Popova et al. defined a unique defect characterized by numerous FTDs and intact HR in the 4% of serous ovarian cancers lacking functional *CDK12*.^20^ The same held true in our mCRPC exome sequencing study, as *CDK12*-mutant tumors constituted a unique mCRPC subtype, genetically distinct from those with other primary genetic drivers, including HRD.^18^

In our mouse model, *Cdk12* loss was sufficient to induce DNA damage, with γH2AX(+) lesions localized to p53 protein expression. Unbiased CRISPR screening further identified a positive relationship between *Trp53* and *Cdk12* loss, while bigenic *Cdk12/Trp53* loss enabled *in vivo* allograft formation capacity not seen with the loss of either gene alone. These data mirror clinical sequencing data—in which *CDK12* and *TP53* inactivation often occurs in the same tumors^18^—and indicate *Cdk12* loss-induced tumorigenesis is enhanced with concomitant inactivation of a compensatory DDR gene. Considering the link between *Cdk12* loss and DNA damage, we first noted AR and MYC signaling increases in the *Cdk12*^*KO*^ organoid model. Upregulation of these pathways is consistent with hypertranscriptive states previously found to promote double-stand breaks.^34^^,^^35^ Interrogation of *Cdk12*^*KO*^ organoids after thymidine block and release demonstrated γH2AX(+) foci during early S-phase, implicating TRCs in double-strand break formation. Indeed, the presence of increased R-loops and direct associations between DNA and RNA Pol-II (based on PLA) support this mechanism. Double-strand breaks generated in this manner may underlie the FTDs previously described in *CDK12*-mutant prostate and ovarian cancer.^18^^,^^20^ While these events did not occur at detectable levels in our organoid system, we suspect they may emerge after longer-term clonal growth. Notably, in *Cdk12*^*KO*^ organoids, DNA damage became persistent in the context of *Trp53* co-ablation—mechanistically underscoring how *Cdk12* and *Trp53* loss synergize to drive tumorigenesis.

Biallelic *CDK12* inactivation in human mCRPC engenders a T cell-predominant immune response potentially driven in part by neo-antigens arising from translated products of FTDs.^18^ In our *Cdk12*^*KO*^ mouse model, preneoplastic prostate lesions were similarly infiltrated by CD4(+) and CD8(+) T cells. Strikingly, a nearly identical infiltrate occurred upon subcutaneous transplantation of *Cdk12*/*Trp53* bigenic mutant organoids into immunocompetent hosts. The composition of the immune infiltrate distinguished this syngeneic model from preexisting Myc-CaP and TRAMP-C2 systems with WT *Cdk12*—both of which induce few CD4(+) and no CD8(+) cells. Indeed, we are unaware of other syngeneic models with significant CD8 infiltration. *Cdk12*/*Trp53*-null allografts may, therefore, be used to study T cell-driven tumor immunity. Sensitivity of these tumors to ICB also opens a promising clinical immunotherapy avenue. Given the absence of FTDs (and consequent neo-antigen formation) in our model, immune response is likely driven by one or more of the numerous inflammatory pathways upregulated in *Cdk12*^*KO*^ organoids. Further exploration of how *Cdk12* loss induces proinflammatory signaling is an important future direction facilitated by our syngeneic model.

Despite its tumor suppressor function, *CDK12* has also been found to promote cell proliferation. For instance, using the CDK12^as^ line employed in our study, Chirackal et al. demonstrated CDK12 promotes G1/S transition by enhancing RNA Pol-II processivity at key DNA replication genes.^49^ Similarly, conditional *Cdk12* ablation in mouse neural progenitors impairs their transit through the cell cycle.^13^ Conversely, elevated CDK12 expression is seen in human malignancies such as HER2(+) breast cancer.^46^^,^^50^ In cell lines derived from these tumors, CDK12-dependent alternative splicing is linked to increased invasiveness and metastatic potential.^51^ Furthermore, CDK12 protein is elevated in gastric cancer and correlates with invasive histology and reduced patient survival.^52^

Choi et al. elucidated a reciprocal interaction between CDK12 and 4E-BP1 that promotes translation of several mTORC1-dependent mRNAs critical for MYC-driven transformation and mitosis.^32^ This report suggests that proliferation of PCa cells dependent on mTOR signaling may—unlike *Trp53*-null cells—exhibit growth inhibition with *Cdk12* loss. We directly tested this premise by co-ablating *Cdk12* in the prostate epithelium of *Pten* knockout mice. Compared with *Pten*-null animals with intact *Cdk12*, these double knockout mice demonstrated improved survival and markedly reduced prostate tumor size, as well as impaired mTOR signaling. These findings align with our previous whole-exome sequencing, which demonstrated *CDK12*/*PTEN* bigenic mutations occur rarely in human mCRPC.^18^

CDK12 and CDK13 are evolutionarily related, structurally similar kinases that phosphorylate the Pol-II CTD to promote transcriptional elongation of overlapping target gene sets.^16^ In leukemia cell lines dual inhibition of both kinases induces genome-wide transcriptional changes and loss of Pol-II CTD phosphorylation—as well as associated proliferation defects and cell death.^17^ These findings are consistent with data from ovarian cancer cell lines showing therapeutic promise for the dual CDK12/13 inhibitor THZ531.^53^^,^^54^ Here, we demonstrate paralog-based synthetic lethality with co-ablation of *CDK12* and *CDK13* in murine organoids and human cell lines. YJ9069, a CDK13/12 degrader, also displayed considerable efficacy in mitigating growth of several mouse-derived cell and organoid lines lacking *Cdk12*—both *in vitro* and *in vivo*. Strikingly, the same premise held in PDXs, as human mCRPC lines with biallelic *CDK12* inactivation also exhibited sensitivity to CDK12/13 degradation. YJ9069 and related agents therefore have promising clinical applicability in *CDK12*-mutant PCa.

Together, our findings define the role of CDK12 in PCa while generating murine models of *Cdk12* loss that recapitulate human disease. *Cdk12* is a tumor suppressor gene responsible for mitigating AR/Myc hypertranscription and TRC-mediated DNA damage. Its inactivation synergizes with *Trp53* loss to drive persistent DNA damage and prostate tumorigenesis associated with T cell infiltration. These data hold potential for near-term clinical impact in patients with *CDK12/TP53*-mutant PCa—in which ICB may elicit an enhanced response. Moreover, CDK13/12- or CDK13-specific inhibitors have strong future potential for treating *CDK12*-mutant PCa.

### Limitations of the study

While we demonstrated upregulated AR signaling and hypertranscription in *Cdk12*-null PCa organoids, further study into mechanisms underlying AR elevation with *CDK12* loss will be fruitful. Similarly, detailed understanding of how *Cdk12* ablation mitigates tumor progression in the setting of *Pten* loss—and the degree to which it represents another form of synthetic lethality—is an important future direction. While *Cdk12* ablation in the mouse prostate induces gene expression alterations and T cell infiltration as seen clinically in *CDK12*-mutant tumors, FTDs characteristic of those tumors are (thus far) undetectable in murine systems. *Cdk12* loss-induced TRCs may contribute to FTD formation with aging, and exploring mechanistic links between these phenomena is of tremendous interest. Finally, we posit reduced CDK13 action underlies antagonistic effects of CDK13/12 inhibitors and degraders on *CDK12-*mutant PCa. While no CDK13-specific inhibitor/degrader currently exists, we surmise such agents would be ideal for effecting paralog-based synthetic lethality in human CDK12-mutant mCRPC.

## Resource availability

### Lead contact

Further information and requests for resources should be directed to the lead contact, Arul M. Chinnaiyan (arul@med.umich.edu).

### Materials availability

All materials used in this paper are available from the lead contact upon request.

### Data and code availability

Sequencing data have been deposited at the National Center for Biotechnology Information Gene Expression Omnibus (NCBI GEO) with the accession number GEO: GSE254390. No custom code was developed in this study. Any additional information required to reanalyze the data reported in this paper is available from the lead contact upon request.

## Acknowledgments

We acknowledge Shuqin Li, Derrick Ekanayake, Fengyun Su, and Rui Wang for technical assistance and Brian Magnuson for sequence analysis support. We thank Lisa McMurry, Amanda Miller, and Christine Caldwell-Smith for histology support, and Dr. Arno Greenleaf (Duke University) for providing the CDK12^as^ line. This study is dedicated to Dr. Nora Navone for her development of the MD Anderson PDX library. Her legacy continues with the efforts of her trainee, Dr. Estefania Labanca. This work was funded by the 10.13039/100000892Prostate Cancer Foundation, National Cancer Institute (NCI) Prostate SPORE Grant (P50-CA186786), NCI Early Detection Research Network (U2C-CA271854), NCI Outstanding Investigator Award (R35-CA231996, A.M.C.), 10.13039/501100001809National Natural Science Foundation of China (22037003, K.D.), and a Programme Grant from Cancer Research UK (DRCRPGNov21y100001, C.J.L.). J.C.-Y.T. is supported by a Department of Defense Prostate Cancer Research Program Idea Development Award (W81XWH-21-1-0458). A.M.C. is a Howard Hughes Medical Institute Investigator, Alfred Taubman Scholar, and American Cancer Society Professor.

## Author contributions

J.C.-Y.T., J.L., J.C., F.Y.F., C.J.L., K.D., and A.M.C. conceived the study and designed the experiments. J.C.-Y.T., Y. Cheng, and P.S. performed all functional experiments, assisted by Y. Chang, S.E., A.P., L.X., and A.J.T. Y.W., D.R.R., and X.C. performed sequencing. X.-M.W. conducted ISH staining. R.M. and S.M. carried out histopathological evaluations. Y.Z., C.C., R.J.R., and M.C. performed bioinformatic analyses. Y.B. conducted immune profiling. J.N., R.B., and S.J.P. performed experiments involving CDK12^as^ cells with supervision from C.J.L. J.C. generated C4-2B *CDK12 KO* lines and performed experiments under supervision of F.Y.F. Y. Chang, J.Y., L.Z., Z.W., X.W., and K.D. contributed to the discovery and synthesis of YJ9069 and YJ5118 compounds. Y.W. and E.L. provided PDX lines. J.C.-Y.T., C.J.L., and A.M.C. wrote the manuscript. S.J.M. reviewed and edited the manuscript.

## Declaration of interests

A.M.C. co-founded and serves on scientific advisory boards (SABs) of Lynx Dx, Flamingo Therapeutics, Medsyn Pharma, Oncopia Therapeutics, and Esanik Therapeutics. A.M.C. is an advisor to Aurigene Oncology Limited, Proteovant, Tempus, Rappta, and Ascentage. C.J.L. received research funding from AstraZeneca, Merck KGaA, Artios, and NeoPhore and consultancy, SAB membership, or honoraria payments from FoRx, Syncona, Sun Pharma, Gerson Lehrman Group, Merck KGaA, Vertex, AstraZeneca, Tango, 3rd Rock, Ono Pharma, Artios, Abingworth, Tesselate, Dark Blue Therapeutics, Pontifax, Astex, NeoPhore, Glaxo Smith Kline, and Dawn Bioventures. C.J.L. has stock in Tango, Ovibio, Hysplex, and Tesselate. C.J.L. is named inventor on patents describing use of DNA repair inhibitors and stands to gain from their development and use. J.C. is an advisor for Exai Bio. F.Y.F. has served on SAB or received consulting fees from Astellas, Bayer, Celgene, Clovis Oncology, Janssen, Genentech Roche, Myovant, Roivant, Sanofi, and Blue Earth Diagnostics. F.Y.F. is also an SAB member for Artera, ClearNote Genomics, Serimmune, and BMS (Microenvironment Division). K.D. is an advisor for Kinoteck Therapeutics and has received financial support from Livzon Pharmaceutical Group. Patents for CDK12/13 degraders/inhibitors used here have been filed by the University of Michigan and Shanghai Institute of Organic Chemistry, with A.M.C., K.D., X.W., J.Y., Y. Chang, and J.C.T. as co-inventors.

## STAR★Methods

### Key resources table

**Table:** 

REAGENT or RESOURCE	SOURCE	IDENTIFIER
**Antibodies**		

Rabbit polyclonal anti-p53	Leica Biosystems	Cat# NCL-L-p53-CM5p; RRID: AB_2895247
Mouse monoclonal anti-tubulin	Abcam	Cat# ab7291; RRID: AB_2241126
Rabbit polyclonal anti-CDK12	Proteintech	Cat# 26816-1-AP; RRID: AB_2880645
Rabbit monoclonal anti-GAPDH (14C10)	Cell Signaling Technology	Cat# 3683 (also 3683S); RRID: AB_1642205
Rabbit monoclonal phospho-Akt (Ser473) (D9E)	Cell Signaling Technology	Cat# 4060; RRID: AB_2315049
Rabbit monoclonal anti-Akt (pan) (C67E7)	Cell Signaling Technology	Cat# 4691; RRID: AB_915783
Rabbit monoclonal anit-S6 ribosomal protein (5G10)	Cell Signaling Technology	Cat# 2217 (also 2217L, 2217S); RRID: AB_331355
Rabbit monoclonal phospho-S6 ribosomal protein (Ser235/236) (91B2)	Cell Signaling Technology	Cat# 4857 (also 4857S); RRID: AB_2181035
Mouse monoclonal anti-vinculin (hVIN-1)	Sigma-Aldrich	Cat# V9131; RRID: AB_477629
Rabbit monoclonal phospho-Rpb1 CTD(Ser2) E1Z3G	Cell Signaling Technology	Cat# 13499; RRID: AB_2798238
Rabbit polyclonal anti-CDK13	EMD Millipore	Cat# EMD Millipore; RRID N/A
Rabbit monoclonal anti-AR (EPR1535(2))	Abcam	Cat# ab133273; RRID: AB_11156085
Mouse monoclonal anti-p63 (4A4)	Abcam	Cat# ab735; RRID:AB_305870
Rabbit polyclonal anti-CDK12	Sigma-Aldrich	Cat# HPA008038; RRID:AB_1078570
Rabbit monoclonal anti-AR	EMD Millipore	Cat# 06–680; RRID:AB_310214
Mouse monoclonal anti-Ki67	BD Biosciences	Cat# 550609; RRID:AB_393778
Rabbit polyclonal anti-CD3	Agilent	Cat# A0452; RRID:AB_2335677
Rabbit monoclonal anti-CD4 (EPR19514)	Abcam	Cat# AB183685; RRID:AB_2686917
Rabbit monoclonal anti-CD8a (D4W2Z)	Cell Signaling Technology	Cat# 98941; RRID: AB_2756376
Rat monoclonal anti-F4/80 (BM8)	Thermo Fisher Scientific	Cat# 14-4801-82; RRID: AB_467558
Mouse monoclonal anti-NK1.1 (PK136)	Thermo Fisher Scientific	Cat# MA1-70100; RRID: AB_2296673
Rat monoclonal anti-cytokeratin 8/18	DSHB	Cat# TROMA-I; RRID: AB_531826
Rabbit monoclonal phospho-Histone H2A.X (20E3)	Cell Signaling Technology	Cat# 9718; RRID: AB_2118009
Rat monoclonal anti-mouse CD31 (390), PE-Cyanine7	Thermo Fisher Scientific	Cat# 25-0311-82; RRID: AB_2716949
Rat monoclonal anti-mouse CD45 (30-F11), PE-Cyanine7	Thermo Fisher Scientific	Cat# 25-0451-82; RRID: AB_2734986
Rat monoclonal anti-mouse TER-119 (CTER-119), PE-Cyanine7	Thermo Fisher Scientific	Cat# 25-5921-82; RRID: AB_469661
Rat monoclonal anti-mouse Ly-6A/E (Sca-1) (Clone D7), PE-Cyanine 7	Thermo Fisher Scientific	Cat# 25-5981-82; RRID: AB_469669
Rat monoclonal anti-mouse CD24 (M1/69), PerCP-eFluor™ 710	Thermo Fisher Scientific	Cat# 46-0242-82; RRID: AB_1834425
Rat monoclonal anti-Cd49f (Integrin alpha6) (eBioGoH3), APC	Thermo Fisher Scientific	Cat# 17-0495-82; RRID: AB_2016694
Rabbit polyclonal anti-PCNA	Abcam	Cat# 18197; RRID:AB_444313
Mouse monoclonal anti-RNA polymerase II, clone CTD4H8	Millipore	Cat# 05–623; RRID: AB_309852
Mouse monoclonal Anti-DNA RNA hybrid S9.6	Millipore	Cat# MABE1095; RRID: AB_2861387
Rabbit polyclonal anti-CK8	Abcam	Cat# ab53280 RRID
Rabbit monoclonal c-Myc antibody	Abcam	Cat# ab32072; RRID: AB_731658
Rabbit monoclonal anti-BRD2 antibody	Bethyl	Cat# A700-008; RRID:AB_2891809
Mouse monoclonal anti-BRD3 antibody	Abcam	Cat# ab50818; RRID:AB_868478
Rabbit monoclonal anti-BRD4 antibody	Bethyl	Cat#A700-004; RRID:AB_2631885
γH2A.X clone JBW301	Millipore	Cat# 05–636; RRID: AB_309864
Anti-CDK12	Abcam	Cat# ab246887; RRID: N/A
β-actin	Santa Cruz	Cat# sc47778; RRID: AB_626632
α-tubulin	Santa Cruz	Cat# 3873S; RRID: AB_1904178
Anti-RNA polymerase II subunit B1 (phospho CTD Ser-2) Antibody, clone 3E10	Millipore Sigma	Cat# 04–1571; RRID: AB_11212363
S9.6 (Kerafast, #Kf-Ab01137–23.0)	Kerafast	Cat#: kf-Ab01137–23.0; RRID: AB_2936195
Anti-DNA-RNA Hybrid Antibody, clone S9.6	Millipore Sigma	Cat#:MABE1095; RRID: AB_2861387
Anti-ssDNA	Sigma-Aldrich	Cat#: ZMS1042; RRID: N/A

**Bacterial and virus strains**		

Ad5 CMV-Cre	This paper	N/A

**Biological samples**		

Patient-derived xenografts (PDX) LTL706B	Vancouver Prostate Cancer	N/A
Patient-derived xenografts (PDX) MDA117	MD Anderson	N/A
Patient-derived xenografts (PDX) MDA153	MD Anderson	N/A
Patient-derived xenografts (PDX) MDA146-12	MD Anderson	N/A
Patient-derived xenografts (PDX) LuCaP23.1	Fred Hutchison	N/A
Patient-derived xenografts (PDX) LuCaP86.2	Fred Hutchison	N/A
Patient-derived xenografts (PDX) LuCaP96	Fred Hutchison	N/A
Patient-derived xenografts (PDX) PC295	Erasmus Medical Center	N/A

**Chemicals, peptides, and recombinant proteins**		

Formaldehyde	Sigma-Aldrich	Cat#F8775
HistoGel	Fisher Scientific	Cat# HG-4000-012
Testosterone pellet	Innovative Research of America	Cat# SA-151
Antigen Unmasking Solution Citrate-Based	Vector Laboratories	Cat# H-3300-250
30% Hydrogen Peroxide	Fisher Scientific	Cat# H325-500
Normal Goat Serum Blocking Solution	Vector Laboratories	Cat# S-1000-20
DAB Peroxidase Substrate kit	Vector Laboratories	Cat# SK-4100
NP-40	Thermo Scientific	Cat#85125
Tween 20	Millipore Sigma	Cat#11332465001
Collagenase Type II, powder	Thermo FIsher	Cat# 17-101-015
Enzalutamide	Selleck Chemicals	Cat# S1250
JQ1	Selleck Chemicals	Cat# S7100
B27 supplement	Gibco	Cat# 17504-044
N-Acetylcysteine	Sigma-Aldrich	Cat# A9165-5g
Recombinant Human EGF	PeproTech	Cat# AF-100-15
Recombinant Human Noggin	PeproTech	Cat# 120-10C
Recombinant Human R-Spondin-1	PeproTech	Cat# 120-38
A83-01	Tocris	Cat# 2939
Recombinant Human FGF-10	PeproTech	Cat# 100-26
Recombinant Human FGF-2	PeproTech	Cat# 100-18C-100ug
Prostaglandin E2 (MW 352.46)	Tocris	Cat# 2296-10 mg
SB202190	Sigma-Aldrich	Cat #S7067-5mg
Nicotinamide	Sigma-Aldrich	Cat# N0636
DHT	Sigma-Aldrich	A8380
Y-27632 2HCL ROCK Inhibitor	Selleck Chemicals	Cat# S1049-10mg
Recombinant Murine EGF	PeproTech	Cat# 315-09
Recombinant Murine Noggin	PeproTech	Cat# 250-38
Recombinant Murine R-Spondin-1	PeproTech	Cat# 315-32
Formalin Buffered 10%	Fisher Chemical	Cat# SF100-4
Ethanol 200 Proof	Sigma-Aldrich	Cat# 64-17-5
Xylene	Leica Biosystems	Cat# 3803665
TryLE Express	Invitrogen	Cat# 12605-010
QIAzol Lysis Reagent	Qiagen	Cat# 79306
YJ9069	This paper	N/A
YJ5118	This paper	N/A
THZ531	Cayman Chemical Company	Cat# 79306
Talazoparib	Selleck Chemicals	Cat# S7048
5,6-Dichlorobenzimidazole 1-beta-D-ribofuranoside	Sigma-Aldrich	Cat# D1916-10MG
Thymidine	Sigma-Aldrich	Cat# T9250-1G
EDTA-free Protease Inhibitor Cocktail	Roche	Cat# 04693159001
PhosSTOP	Roche	Cat# 04906837001
Matrigel® Growth Factor Reduced (GFR) Basement Membrane Matrix	Corning	Cat# 356230
Lipofectamine™ 3000 Transfection Reagent	Invitrogen	Cat#L3000001
Lipofectamine™ RNAimaxTransfection Reagent	Invitrogen	Cat#13778150
Puromycin	Thermo Scientific	Cat#A1113803
Blasticidin	Thermo Scientific	Cat#A1113903
Fast SYBR™ Green Master Mix	Thermo Scientific	Cat#4385612

**Critical commercial assays**		

CellTiter-Glo® Luminescent Cell Viability Assay	Promega	Cat#G7572
VECTASTAIN® Elite Avidin-Biotin Complex (ABC)-HRP Detection Kit, Peroxidase (Standard)	Vector Laboratories	Cat# PK-6100
CellTiter-Glo® 3D Luminescent Cell Viability Assay	Promega	Cat#G9683
10X Genomics Chromium Single Cell 3′ Library Gel bead Kit V3.1	10X Genomics	N/A
Maxima First Strand cDNA Synthesis Kit for RT-qPCR	Thermo Fisher Scientific	Cat# K1641
Pierce 660nM Protein Assay Reagent	Thermo Fisher Scientific	Cat# 22660

**Deposited data**		

Raw and analyzed data	This paper	GEO: GSE254390

**Experimental models: Cell lines**		

Mouse prostate organoids	This paper	N/A
Myc-CaP	ATCC	N/A
TRAMP-C2	ATCC	N/A
C4-2B	ATCC	N/A
CDK12^as^ HeLa	This paper	N/A

**Experimental models: Organisms/strains**		

Mouse: B6.129-Cdk12 tm1Fmj/Narl	National Laboratory Animal Center	N/A
Mouse: Tg(Pbsn-cre)4Prb/J	The Jackson Laboratory	JAX: 026662; RRID: IMSR_JAX:026662
Mouse: B6.129S4-Ptentm1Hwu/J	The Jackson Laboratory	JAX: 006440; RRID:IMSR_JAX:006440
Mouse: C57BL/6J	The Jackson Laboratory	JAX: 000664; RRID:IMSR_JAX:000664
Mouse: FVB/NCrl	Charles River Laboratory	#207
Mouse: NOD Cg-Prkdc<scid> ll2rg<tm1Wjl>SzJ	The Jackson Laboratory	JAX: 005557; RRID:IMSR JAX:005557
Mouse: CB17/Icr-Prkdcscid/IcrIcoCrl	Charles River Laboratory	Cat#236

**Oligonucleotides**		

Cdk12 (Mm01306742_m1)	Life Technologies	Cat#4331182
Hprt (Mm00660704_m1)	Life Technologies	Cat#4331182
Primers for Trp53 and its target genes, see Table S3	This paper	N/A
sgRNA sequences, see Table S3	This paper	N/A
Human CDK12 sgRNA CTTGGTATCGAAGCACAAGC	This paper	N/A
Human CDK12 sgRNA ACTTTGCAGCCGTCATCGGG	This paper	N/A

**Recombinant DNA**		

MusCK Library	–	Addgene Plasmid #174196
LentiCRISPRv2 Plasmids	–	Addgene Plasmid #107402
PX458 plasmid	–	Addgene Plasmid #48138

**Software and algorithms**		

FCS Express 7	This paper	https://denovosoftware.com/
MAGeCK (version 0.5.9.5)	Li et al.^55^	https://hpc.nih.gov/apps/MAGeCK.html
10X Genomics Cell Ranger pipeline (v5.0)	This paper	https://www.10xgenomics.com/support/software/cloud-analysis/latest/miscellaneous/CA-supported-products
R Package Seurat (v4.1)	Hao et al.^56^	N/A
R Package scDblFinder	Germain et al.^57^	N/A
MsigDB	Liberzon et al.^58^	N/A
ImageJ	–	https://imagej.net/ij/
SoupX	Young et al.^59^	N/A
bowtie2(version 2.4.5)	Langmead et al.^60^	N/A
Cutadapt	Kechin et al.^61^	N/A
BWA-MEM	Li et al.^62^	N/A
Limma function loessfit	Ritchie et al.^63^	N/A
DNACopy	Seshan et al.^64^	N/A
CNVEX	Chowdhury et al.^65^	N/A

### Experimental model and study participant details

#### Cell lines

C4-2B, TRAMP-C2, and Myc-CaP lines were obtained from the ATCC. C4-2B cells were cultured in RPMI with 10%FBS. TRAMP-C2 cells were cultured in Gibco DMEM with 5% FBS, 5% Nu-Serum, 4mM Glutamine, 5 μg/ml human insulin and 10nM DHT. Myc-CaP cells were cultured in Gibco DMEM GlutaMax with 10% FBS. CDK12^as^ cells were provided by Dr Arno Greenleaf, Duke University.

#### Mouse and PDX models

*Cdk12*^*f/f*^ mice (B6.129-*Cdk12* ^*tm1Fmj*^/Narl) were obtained from the National Laboratory Animal Center (Taiwan, R.O.C.). *Probasin-Cre* (Stock#026662), *Rosa*^*mT/mG*^(Stock#007676), and *Pten*^*f/f*^ (Stock#006440) mice were obtained from the Jackson Laboratory (Bar Harbor, ME). The animals were interbred, backcrossed, and maintained on a C57Bl/6J background. For syngeneic models, male C57Bl/6J (Stock# 000664) mice (6-8-weeks old) were obtained from the Jackson Laboratory, and male FVB (Stock #207) mice were obtained from Charles River Laboratory (Wilmington, MA). NOD Cg-Prkdc<scid> ll2rg<tm1Wjl>SzJ (NSG) mice were obtained from the Jackson Laboratory, (Stock#005557) while CB17SCID mice were obtained from Charles River (Stock#236). All animals were housed in pathogen-free containment with a 12-h light-dark cycle and *ad libitum* food and water. The University of Michigan Institutional Animal Care and Use Committee (IACUC) approved all animal studies. The LTL706B (*CDK12*-mutant) tumor was obtained from the Vancouver Prostate Center and initially established in the renal capsule of NSG mice with a testosterone pellet (12.5 mg) implant. Once tumors grew successfully, we transferred them into subcutaneous pockets of CB17SCID mice for therapy studies. Other PDX, such as MDA117, 328 (*CDK12*-mutant) and MDA153, 146-12 (*CDK12*-intact), were obtained from MD Anderson. LuCaP23.1, 86.2, and 96 (*CDK12*-intact) PDX tumors were obtained from the University of Washington. Tumors from MDA and LuCaP PDX lines were maintained subcutaneously in dorsal flanks of CB17SCID male mice. The PC295 (*CDK12*-intact) PDX line was obtained from Erasmus Medical Center, Rotterdam, the Netherlands.^66^

#### Organoid models

Mouse prostates were harvested from 52-week-old mice, and single cell isolation was adapted from previously published protocols.^31^^,^^67^ First, prostates were digested with 1 mg/mL collagenase Type II (Gibco) for 1 h at 37°C followed by TryLE (Gibco) digestion. After TryLE digestion, samples were inactivated with an excess of DMEM containing 10% fetal bovine serum (FBS), and samples were sequentially passed through 100 μm and 40 μm cell strainers to remove debris. Flow cytometry analysis used established marker profiles.^31^ Briefly, fresh cells were incubated in PBS with fluorophore-conjugated antibodies at dilutions indicated in Table S3 for 30 min at 4°C. DAPI was added for the final 5 min of the incubation to act as a dead cell marker. Cells were analyzed on MoFlo Astrios EQ running Summit software (version6.3; Beckman-Coulter, Brea, CA). Gates were established using fluorescence minus one approach, and plots were generated in FCS Express 7 (*DeNovo* Software). The Flow Cytometry Core from the University of Michigan assisted with the flow sorting experiment. Isolated prostatic epithelial cells were embedded in 50-μL drops of Matrigel and overlaid with mouse prostate organoid medium. Media was changed every 2–3 days and organoids were passaged on a weekly basis. Prostate organoid cells were seeded at 1000 cell density in a matrix dome on Day 0 in medium without EGF or DHT. Cell viability was assayed starting at Day 1 for 6 days according to the CellTiter-Glo 3D kit (Promega G9683). For the antiandrogen response assay, the procedure was adapted from a previously published protocol.^68^ Briefly, organoids were seeded at 2000 cells on Day 0 in media minus EGF, with 1 nM DHT or 10 μM of enzalutamide (Selleck Chemicals) added. For JQ1 treatment, 1 μM concentration was use in complete media. Cell growth was assayed on Day 7 using the CellTiter-Glo 3D kit. Organoid allograft models were generated by subcutaneous injection of Matrigel-suspended organoid cells (3 x 10^6^ cells per injection) into dorsal flanks of NSG mice. Animals were monitored for tumor growth weekly. Once *Cdk12*^*KO*^-sgp53 tumors reached 1000 mm^3^, the tumors were resected, cut into small chunks, and subcutaneously implanted into both flanks of C57Bl/6J mice for generation of the syngeneic model.

### Method details

#### Histological analysis and immunohistochemistry

Prostate tissue and allograft tumors were fixed in formalin overnight, dehydrated with ethanol, and paraffin embedded. Five-μm-thick sections were prepared for H&E staining and immunohistochemistry. Two pathologists with expertise in genitourinary evaluated the H&E-stained formalin-fixed paraffin-embedded (FFPE) tissue sections in a blinded manner. Before the assessment, four histopathological scoring schemas were created based on the temporal progression of prostate pathology. These categories were: Category 0: Normal prostatic epithelium; Category 1: Epithelial hyperplasia; Category 2: Focal high-grade prostatic intraepithelial neoplasia (PIN); Category 3: Florid high-grade PIN/atypical intraepithelial neoplasm (AIP) and intraductal carcinoma. Each prostate sample was evaluated for overall percent prevalence for each of these four categories. Immunohistochemistry was performed manually or using the Ventana automated slide staining system (Roche-Ventana Medical System). Antibody concentrations are listed in Table S3. For immunohistochemical staining of organoids, organoids were embedded in Histogel and fixed with 4% PFA for 1h, then ethanol dehydrated, and paraffin embedded. For manual staining procedure, samples were then deparaffinized and incubated in Antigen Unmasking Solution (Vector Laboratories, H-3300). Endogenous peroxidases were inactivated via incubation in 3% hydrogen peroxide (Sigma). Primary antibodies were diluted in 10% normal goat serum with overnight incubation; and antibody detection was achieved with species-specific VECTSTAIN Elite ABC kits (Vector Labs) and DAB Peroxidase Substrate kit (Vector Labs).

#### RNA *in situ* hybridization

*Cdk12* gene expression was detected in FFPE tissue sections using the RNAscope 2.5 HD Brown kit (Advanced Cell Diagnostics, Newark, CA) and the target probe against the mouse *Cdk12* gene (cat # 444881). The *Cdk12* target probe is complementary to NM_02695.2, 102-1021nt. RNA quality was evaluated by a positive control probe against mouse low-copy housekeeping gene (*ppib*). Assay background was evaluated by a negative control probe targeting bacterial *DapB* gene. FFPE tissue blocks were cut into 4μm sections. The tissue sections were baked at 60°C for 1 h, deparaffinized in xylene, and dehydrated in 100% ethanol followed by air drying. After hydrogen peroxide pretreatment and target retrieval in citrate buffer at 100°C, tissue sections were permeabilized using protease and hybridized with the target probe in the HybEZ oven for 2 h at 40°C, followed by a series of signal amplification steps. Finally, the sections were chromogenically stained with DAB and counterstained with 50% Gill’s Hematoxylin I (Fisher Scientific, Rochester, NY).

#### Immunofluorescence

Immunofluorescence (IF) was performed on 5-μm-thick FFPE tissue sections using anti-TP63 mouse monoclonal antibody (1:100; Abcam, catalog no. ab735), anti-CK8 rabbit polyclonal antibody (1:100, Abcam, catalog no. ab53280), anti-p53 rabbit polyclonal antibody (Leica, cat no. P53-CM5P-L), and anti-CDK12 rabbit polyclonal antibody (Atlas, cat no. HPA008038). TP63/CK8 double IF was carried out on a Discovery Ultra automated slide staining system (Roche-Ventana Medical Systems) using CC1 95°C for antigen retrieval, followed by primary antibody (anti-TP63) incubation, OmniMap anti-mouse horseradish peroxidase (HRP), and signal development using the Discovery Cy5 Kit (RTU, Roche-Ventana Medical Systems, catalog no. 760-238). Secondary antibody staining was performed with heat denaturation before the second primary antibody (anti-CK8) incubation, OmniMap anti-rabbit HRP kits, and signal development using Discovery FITC (RTU, Roche-Ventana Medical Systems, catalog no. 760-232). With a similar algorithmic process, p53/CDK12 double IF was performed first with p53 IF with Cy5, followed by CDK12 IF with FITC. The staining was independently assessed by three study participants including one pathologist (J. Tien, Xiao-Ming Wang, and R. Mannan) at ×100 and ×200 magnification to assess for presence and pattern of expression. For R-loop staining, organoids were plated as 2D cells on coverslips and incubated overnight at 37 in a 5% CO2 incubator. Cells were treated with ice-cold, 100% methanol for 20 min at −20°C and permeabilized with 0.5% Triton X-100 for 10 min. Cells were incubated with S9.6 antibody (Sigma-Alrich; no. MABE1095) at 1:50 dilution overnight at 4°C, followed by secondary anti-mouse IgG conjugated with Alex Fluor 594 for 1 h at room temperature. For negative control, cells were incubated with RNase H for 4 h before primary antibody incubation. The nuclear fluorescence intensity of R-loop per cell was determined with ImageJ software.

#### Adenoviral Cre and CRISPR/Cas-9 lentiviral transduction

Adenoviral Cre-mediated recombination of *Cdk12* in mouse prostate organoids was performed by adenoviral delivery of CRE recombinase as previously described.^69^ Similarly, CRISPR/Cas-9 mediated knockout of *Trp53*, *Pten*, and *Cdk13* was performed by lentiviral delivery of plasmids encoding Cas9 and gRNA sequences using LentiCRISPRv2 plasmids. sgRNA sequences are listed in Table S3.

#### *In vivo* CRISPR screening

The MusCK library was a gift from Xiaole Shirley Liu (Addgene 174196). The MusCK library contains guide RNAs targeting 4922 mouse genes that are implicated in cancer. A total of 10ˆ7 *Cdk12*^*KO*^ organoids were transduced with lentivirus containing the MusCK library at a multiplicity of Infection (MOI) of 0.3 to achieve about 100x coverage. After puromycin selection for 5 days, ∼30% of the surviving cells were stored as Day0 input samples at −80°C, and the remaining cells were cultured for *in vivo* screening. 3 x 10^6^ cells were prepared for each injection site for a total of 10 injection sites. Animals were monitored every week for tumor growth. Resulting tumors were harvested for genomic DNA extraction. PCR and purification of the regions containing the sgRNA were performed to generate the sequencing library. Each library was sequenced at approximately 3 million reads. Cutadapt^61^ was used to trim reads to the bare sgRNA sequences. The trimmed reads were then aligned to a reference built from the sgRNA sequences in the library using bowtie2(version 2.4.5).^60^ Finally, MAGeCK (version 0.5.9.5)^55^ was used to quantify sgRNAs.

#### RNA isolation and quantitative real-time PCR

Total RNA was isolated using QIAzol Lysis Reagent (QIAGEN), and cDNA was synthesized following Maxima First Strand cDNA Synthesis Kit (Thermo Fisher Scientific) instructions. Quantitative real-time PCR (qPCR) was performed in triplicate using either ThermoFisher Taqman Gene Expression assay or standard SYBR green protocols using SYBR Green PCR Master Mix (Applied Biosystems) on a QuantStudio 5 Real-Time PCR system (Applied Biosystems). The target mRNA expression was quantified using the ΔΔCt method and normalized to the expression of the housing keeping gene. Primer sequences and Taqman probes are listed in Table S3.

#### Compounds

YJ9069 and YJ5118 were synthesized in Dr. Ke Ding’s lab.^70^ THZ531 was purchased from Cayman Chemical Company or Selleckchem. 1NM (aka 1NM-PP1) was purchased from Axon Medchem. Talazoparib was purchased from Selleckchem. 5,6-dichloro-1-beta-D-ribofuranosylbenzimidazole (DRB) and Thymidine were purchased from Sigma-Aldrich.

#### Drug treatment of organoids and cell lines

To generate drug response curves, mouse organoids were digested with TryPLE for 10 min at 37°C, dissociated into single cells, and neutralized with FBS. Cells were resuspended in 20% Matrigel, plated in triplicate at a seeding density of 5000 cells/well in 48-well microplates. The next day, 8 doses of YJ9069 were dispensed at 3-fold dilution from 0.01 μM to 10 μM. Cell viability was assayed after five days using luminescence measurement via CellTiter-Glo 3D (Promega G9683). Drug response curves were generated by nonlinear regression representing percentage of viable cells versus log drug concentration using Graphpad Prism 9. IC_50_ values were calculated by the equation log(inhibitor) versus response (variable slope, four parameters). Two-way ANOVA was used to compare dose-response curves. A similar method was used to determine drug response of PDX organoids and cell lines.

#### ICB treatment of mice

Tumor-bearing mice were injected intraperitoneally every four days with either cocktail of anti-PD1 (250 μg/dose, #BE0146, BioXcell) and anti-CTLA4 (100 μg/dose, #BE0131, BioXcell) or control IgG (350ug/dose, #BE0089 and BE0087, BioXcell). Tumors were measured with calipers twice a week. On day 18, mice were euthanized, and tumors were collected for immunoprofiling.

#### Immunoprofiling of T cells

Resected tumors were cut into small pieces using spring scissors and digested in 0.5 mg/mL collagenase D (Roche: cat#: COLLD-RO) and 0.25 mg/mL DNase I at 37°C for 30 min. After digestion, samples were passed through 70 μm cell strainers followed by ficoll density gradient centrifugation (Lymphoprep: STEMCELL; cat# 07851). After removing erythrocytes, mononuclear cells were stimulated with phorbol 12-myristate 13-aetate (PMA), ionomycin, brefeldin A, and monensin in the T Cell-medium for 4 h at 37°C. Cells were then blocked with anti-mouse CD16/32 (Biolegend; cat# 550994) at room temperature for 1 min, then stained with anti-CD90 (BioLegend; cat# 140327), anti-CD8 (BD Biosciences; cat# 560776), and anti-CD4 for 8 min in the dark. After staining, cells were washed and fixed/permeabilized using Perm-Fix buffer. Subsequently, cells were stained for anti-Ki67 (Thermo Fisher Scientific; cat# 56-5698-82), anti-TNFα (BioLegend; cat#506324), anti-IFN-γ (BD Biosciences; cat# 563773), and anti- Granzyme-B for 10 min. After further washing, the cells were analyzed on the BD LSRFortessa Cell Analyzer, and flow cytometry data were analyzed using FlowJo V10.8.1.

#### Drug treatment of mice

The anti-tumor efficacy of YJ9069 was evaluated in various subcutaneous xenografted and allografted models. In each case, when tumors reached ∼100–200 mm^3^, mice were randomized into two groups of 6–10 mice. Each group received either YJ9069 (15 mg/kg or 30 mg/kg) or vehicle (2 times/week) by IV injection for 14–30 days. Tumor volume was measured twice weekly by caliper following the formula (π/6)(LxW^2^) where L and W are the length and width of the tumors. At the end of the time course, tumors were excised, weighed, and collected for histological analysis.

#### Immunoblotting

Cells were pelleted and lysed using 1X cell lysis buffer (Cell signaling, Cat# 9803S) with EDTA-free Protease Inhibitor Cocktail (Roche, Cat# 4693159001) and PhoSTOP (Roche, Cat# 04906837001). Protein concentration was determined using Pierce 660 nM Protein Assay Reagent (Thermo Fisher Scientific, Cat# 22660), and 20–30 μg of total protein was loaded in each lane. Proteins were separated by NuPAGE 3–8% or 4–12% Tris-Acetate Midi Gel (Invitrogen, Cat# WG1402BX10) and transferred to nitrocellulose membranes (Fisher, Cat# 88018). Membranes were blocked with 5% non-fat dry milk/PBS for 1 h and then incubated with primary antibody overnight at 4°C. The primary antibody information is listed in Table S3. After three washes with 1 X TBS (ThermoFisher, Cat J75892-K8 pH7.4) containing 0.1% Tween 20 (Sigma, Cat P9416-100mL), membranes were incubated with 1:3000 diluted horseradish peroxidase (HRP) labeled secondary antibodies in 5% milk/PBS for 2 h at room temperature. After three washes with TBST, membranes were imaged using an Odyssey CLx Imager (LiCOR Biosciences). For the analysis of CDK12^as^ cells, anti-human antibodies were used for the following proteins: CDK12 (Cell Signaling 11973S, Abcam ab246887), β-Actin (Santa Cruz sc47778), α-TUBULIN (Santa Cruz 3873S), RNA Pol-II subunit B1 phosphor CTD Ser-2 Antibody, (clone 3E10, Millipore 04–1571). For CDK12^as^ cells, lysates were harvested with NP250 buffer (20 mmol/L Tris, pH 7.6, 1 mmol/L EDTA, 0.5% NP40, 250 mmol/L NaCl) containing protease inhibitor cocktail tablets (Roche). Samples were run alongside a Chameleon Duo protein ladder and transferred to nitrocellulose membranes, blocked using LICOR TBS blocking buffer (927–50000), developed using LICOR IRDye secondary antibodies, and imaged using an Odyssey CLx.

#### CDK12^as^ survival assays

For siRNA experiments, cells were reverse transfected using Lipofectamine RNAimax Transfection Reagent (Promega) in 6-well plates for 24 h prior to splitting to final destination plates. For CDK12 cDNA experiments, cells were forward transfected with Lipofectamine 2000 Transfection Reagent in 6-well plates. After 24 h, cells were divided into destination 6 well plates. 24 h after seeding into destination 6-well plates, media containing small molecule inhibitors (talazoparib or 1NM) was added and replenished twice per week. After 2 weeks, colonies were washed with PBS, fixed with 10% trichloroacetic acid, and stained with sulforhodamine B. Image scans of stained colonies were analyzed for colony number and growth area by thresholding a grayscale image followed by conversion to a binary image with a watershed algorithm applied within ImageJ.

#### CDK12^as^ γH2AX and Rad51 analysis

Cells were seeded in 96-well plates for 24 h, exposed to indicated drug combinations for an additional 24 h, or exposed to 10 Gy γ irradiation for 15 min. Cells were fixed with 4% PFA for 1 h at room temperature and washed twice with PBS. Permeabilization was performed with 0.2% Triton x100 in PBS and blocked using a PBS solution with 1% BSA and 2% Fetal Bovine Serum. Primary antibody incubation (γH2AX using clone JBW301, Millipore 05–636 or RAD51 detection using Abcam ab63801) was carried out overnight at 4°C, and secondary antibody incubations were carried out for 40 min at room temperature. DAPI stain was added 10 min prior to development. Immunofluorescence was detected on an ImageXpress high content spinning disc microscope, and the number of foci per cell was determined with metaXpress software.

#### siRNA screening and transfection

CDK12^as^ cells (1000 cells/well) were reverse transfected in a 96 well plate format with a custom siGENOME SMARTPool (Dharmacon) siRNA library—including genes involved in mRNA splicing and/or control of intronic truncating mutations (two processes in which CDK12 dysfunction has been implicated^10^), genes whose expression is dysregulated in *CDK12* mutant ovarian or PCa,^4^^,^^18^^,^^19^ genes that encode putative CDK12-interacting proteins,^71^ and genes that encode likely CDK12 phosphorylation targets^72^—as previously described^73^ using Lipofectamine RNAimax Transfection Reagent (Promega). Positive (siPLK1) and negative controls (siCON1, Dharmacon) were also included in each plate. After 24 h, media was replaced with new media containing 1NM (0.3 μM) or vehicle (DMSO), then cells were continuously cultured for six days further, at which point cell viability was estimated by the addition of CellTiter-glo reagent to the media for 10 min. Drug Effect Z scores were calculated from the resultant luminescence data as described previously.^74^ Each screen was carried out in triplicate, with the data being combined in the final analysis. For single gene siRNA experiments, C4-2B cells were plated in 96-well plates and allowed to adhere overnight. The next day, cells were transfected using siGENOME SMARTPool (Dharmacon) against the indicated genes (CCNK, CDK13) or non-targeting control (NTC) as above. The plate was then placed in an IncuCyte S3 (Sartorius) and cell growth monitored over the indicated time frame.

#### Generation of CRISPR knockout of *Cdk12*/*CDK12* in Myc-CaP cells and C4-2B

Short guide RNAs targeting the exons of mouse *Cdk12* were designed by Benchling (https://www.benchling.com/). Non-targeting control sgRNA and Cdk12-sgRNAs were cloned into lentiCRISPR v2 plasmid (Addgene 98290), and the sgRNA sequences are listed in Table S3. Myc-CaP cells were transiently transfected with control sgNT or pair of two independent *Cdk12*-targeting sgRNAs. Twenty-four hours after transfection, cells were selected with 10 μg/mL puromycin for three days. Immunoblot was performed to detect knockout efficiency. Individual cells were isolated to generate monoclonal lines for analysis of knockout by Immunoblot. More than 100 clones were screened in this process. For C4-2B knockouts, cells were transfected with the PX458 plasmid (Addgene 48138) containing the guide sequence (CTTGGTATCGAAGCACAAGC or ACTTTGCAGCCGTCATCGGG) targeting exon 1 of *CDK12* using Lipofectamine 3000 (Thermo Fisher) according to manufacturer’s instructions. Approximately 48–72 h after transfection, cells were sorted for green fluorescence protein (GFP) into single cells in a 96-well plate format. Clones were expanded and validated by Western blot and sequencing for the target site. Approximately 50 clones were screened in this process.

#### Colony formation assay

Cells were seeded into six-well plates (1x10^4^ cells/well) and allowed to grow for 5 days in complete medium. They were then fixed in 10% formalin for 30 min at RT and stained for 30 min in crystal violet (Fisher Chemical, C581-100) diluted to 1% by volume in H_2_O. Following H_2_O washes, samples were dried overnight and imaged on an Epson Perfection V33 scanner.

#### R-loop detection using dot-blot

5x10^6^ cells were collected and resuspended in 600μL pH8.0 Tris-EDTA buffer. After addition of 37.5μL 20% SDS (Ambion, AM9820) and 30μL 20 mg/ml Proteinase K (Qiagen, # 19133), samples were digested overnight at 56°C. Subsequently, 600μL phenol/chloroform/isoamyl alcohol (25:24:1) pH 8.0 (Fisher Scientific, #327111000) was added for DNA extraction. DNA was then precipitated with 0.1x volume NaAc (Sigma-Aldrich, S7899) and 2.5x volume ethanol. Purified genomic nucleic acids were dissolved in 10mM Tris-HCl pH8.0. After quantification, genomic nucleic acids samples were digested at 37°C for overnight using a restriction enzyme cocktail of BsrgI, EcoRI, HindIII, SspI, and XbaI in Buffer r2.1 (NEB, #B6002S), followed by incubation with RNase A and RNAse III for 3 h to remove both single-strand and double-strand free RNA. After digestion, enzymes were inactivated at 65°C for 20 min. As a negative control, half the digested genomic DNA of each sample was treated with RNase H (NEB) at 37°C overnight and then inactivated at 65°C for 20 min. 200ng DNA of each sample was spotted onto 6XSSC pre-wetted NC membrane (Thermo Scientific, #88018) using a slot blot apparatus (BioRad, # 1706545) and vacuum suction. As a separate loading control, the same amount of DNA was denatured in 0.5M NaOH, 1.5M NaCl at 95°C for 10 min, then neutralized in 1M NaCl, 0.5M Tris-HCl pH 7.4 at room temperature for 10 min prior to spotting as described above. Spotted membranes were UV crosslinked (0.12J/m2) and then blocked in 5% milk/PBS. Membranes were incubated overnight with either S9.6 (Kerafast, #Kf-Ab01137–23.0) or ssDNA (Sigma-Aldrich # ZMS1042) antibodies, then washed and incubated with anti-rabbit or anti-mouse secondary-HRP antibodies (Bio-Rad, #1706515 and #1706516) for 1h hour at RT. After three washes with TBST, membranes were exposed to ECL (Thermo Scientific, #34095) and imaged using the Odyssey CLx Imager (LiCOR Biosciences).

#### *In situ* proximity ligation assay (PLA)

Organoids were fixed with 4% PFA at RT for 10min and washed with PBS three times. Fixed organoids were then dehydrated through an ethanol series, embedded in paraffin, and cut into 4-μm-thick sections. Prior to PLA staining, slides containing sections were deparaffinized, rehydrated, and boiled for 15 min in citrate buffer (pH 6.0) for antigen retrieval. After cooling, slides were washed in PBS and permeabilized with 0.5% Triton X-100% (in PBS) for 10 min. The remainder of the staining protocol was performed using the NaveniFlex PLA kit with some modifications. Briefly, slides were blocked with 10% goat serum for 1 h at RT and slides were then incubated with the desired primary antibodies at 4C overnight before completion of staining as per manufacturer’s instructions. Finally, after staining, slides were mounted with Prolong Gold Antifade mounting media. The number of foci per cell was determined with ImageJ software.

### Quantification and statistical analysis

#### Single cell RNA sequencing (scRNA-seq) and data analysis

scRNA-seq for dissociated mouse prostate tissues and organoids was performed using 10X Genomics Chromium Single Cell 3′ Library Gel bead Kit V3.1 according to the manufacturer’s protocol. The libraries were sequenced with the Illumina Hiseq 2500 or NovaSeq 6000 according to recommended specifications. After sequencing, read demultiplexing, alignment, and gene quantification were conducted with the 10X Genomics Cell Ranger pipeline (v5.0) and the pre-built mouse reference genome (mm10). For libraries from mouse prostate tissues, custom reference genome including sequences of GFP and tdTomato were used. Downstream analyses using the filtered gene count matrix were performed with R package Seurat (v4.1)^56^ if not specified otherwise. Low quality cells were further filtered based on total UMI, number of detected genes, and fraction of mitochondrial reads per cell using the Outlier function from the scatter package^75^; specifically, cells that were three times of mean absolute deviation (MAD) away from median on the three metrics were removed. In addition, putative doublets were identified with the R package scDblFinder^57^ and removed. After cell filtering, mitochondrial genes were also removed from the matrix. SoupX^59^ was used to adjust the count matrix in order to minimize impact of ambient RNA. After all QC steps, the SoupX corrected count matrix was then normalized using the NormalizeData function with the "LogNormalize" method. The top 2000 highly variable genes were then identified with FindVariableFeatures with the “vst” method, followed by ScaleData, RunPCA, and RunUMAP steps to obtain a 2-D map of the cells. The FindNeighbors and FindCluster functions were used to assign cells into clusters. Cell annotation was based on prediction using the TransferData method and a public dataset as ref. ^76^. RNA velocity analysis on the *Cdk12*^*WT*^ organoid was conducted with velocyto^77^ to count spliced and unspliced RNA and scvelo^78^ to calculate RNA velocity and pseudotime and visualize velocity vector field as streamlines. Cells from *Cdk12*^*KO*^ organoids were projected into UMAP of *Cdk12*^*WT*^ and annotated using the MapQuery function of Seurat. To conduct GSEA between *Cdk12*^*KO*^ and *Cdk12*^*WT*^, pseudo-bulk gene expression profiles were generated by summing counts for each cell type in *Cdk12*^*KO*^ and *Cdk12*^*WT*^, respectively; normalized expression in TPM was then calculated with edgeR by incorporating TMM scaling factors.^79^ Genes ranked by logFC were used as input for pre-ranked GSEA with fgsea.^80^ Hallmark gene sets were downloaded from MsigDB.^81^ The human CDK12 gene signature (hCDK12.DN) was defined using common genes down-regulated in PCa patients with *CDK12* mutation and siCDK12 knockdown LNCaP cells.^18^ The mouse homolog genes of the human CDK12 signature were mapped using biomaRt^82^

#### RNA-seq and data analysis

RNA extraction was followed by ribosomal RNA (rRNA) depletion. The rRNA-depleted RNA libraries were prepared using the KAPA RNA HyperPrep Kit (Roche) and subjected to the Agilent 2100 Bioanalyzer for quality and concentration. Transcripts were quantified by alignment-free approach kallisto^83^ using index generated from mouse reference genome (mm10) and then summed to obtain gene level counts. Differential analysis was performed using limma-voom procedure^63^^,^^84^ after TMM-normalization^85^ of gene level counts with calcNormFactors of edgeR.^79^ Genes with mean Transcripts Per Million (TPM) less than 1 in both control and treatment groups were considered as lowly expressed genes and excluded for differential analysis. Enrichment of Hallmark gene sets downloaded from MSigDB^58^ were examined with fgsea^80^ using genes ranked by logFC estimated from limma as input.

#### Whole-genome sequencing

Whole-genome sequencing was performed as per our standard protocols.^18^ Briefly, tumor genomic DNA was purified using the AllPrep DNA/RNA/miRNA kit (Qiagen). *Cdk12*^*WT*^ (reference genome) and *Cdk12*^*KO*^ organoid-derived DNAs were sequenced on the Illumina NovaSeq 6000. Short reads were trimmed off sequencing adapters and aligned to the GRCm38 reference genome using BWA-MEM,^62^ with settings "-Y -K 10000000″, duplicates were removed per Picard^86^ rules, and depth of coverage was calculated using Mosdepth,^87^ with settings "-x -F 1796″, excluding unmapped, not primary, QC-failed, and duplicate reads. Average depth of coverage in 10kb bins was normalized per sample to the total sequencing depth and adjusted for GC-bias using weighted LOWESS as implemented in Limma function loessfit.^63^ The resulting coverage profiles were masked for outliers and segmented using CBS as implemented in DNACopy.^64^ The resulting segmentation profiles were pruned using CNVEX as described previously.^65^ The presence of focal gains was determined by identifying segments >50kb in size with a normalized log-coverage of >0.5. The lack of FTDs was visually confirmed through visualizations of coverage profiles using R/ggplot2 and IGV.

## Supplemental information

Document S1. Figures S1–S7

Table S1. List of sgRNA fold changes from the *in vivo* CRISPR screen, related to Figure 3

Table S2. siRNAs used for synthetic lethality screening in CDK12^as^ cells, related to Figure 6

Table S3. Antibody information, qPCR primer sequences, and sgRNA sequences, related to STAR Methods

Document S2. Article plus supplemental information
