# Supplementary material for: CDK12 loss drives prostate cancer progression, transcription-replication conflicts, and synthetic lethality with paralog CDK13
Source: Cell Rep Med. 2024 Oct 4;5(10):101758. doi: 10.1016/j.xcrm.2024.101758 (PMC11513839; doi:10.1016/j.xcrm.2024.101758)
Supplement: Document S1. Figures S1–S7 [file mmc1.pdf]

Cell Reports Medicine, Volume *5*

Supplemental information

CDK12 loss drives prostate cancer progression, transcription-replication conflicts, and synthetic lethality with paralog CDK13

Jean Ching-Yi Tien, Jie Luo, Yu Chang, Yuping Zhang, Yunhui Cheng, Xiaoju Wang, Jianzhang Yang, Rahul Mannan, Somnath Mahapatra, Palak Shah, Xiao-Ming Wang, Abigail J. Todd, Sanjana Eyunni, Caleb Cheng, Ryan J. Rebernick, Lanbo Xiao, Yi Bao, James Neiswender, Rachel Brough, Stephen J. Pettitt, Xuhong Cao, Stephanie J. Miner, Licheng Zhou, Yi-Mi Wu, Estefania Labanca, Yuzhuo Wang, Abhijit Parolia, Marcin Cieslik, Dan R. Robinson, Zhen Wang, Felix Y. Feng, Jonathan Chou, Christopher J. Lord, Ke Ding, and Arul M. Chinnaiyan

Supplemental Information

CDK12 Loss Drives Prostate Cancer Progression, Transcription-Replication Conflicts, and Synthetic Lethality with Paralog CDK13

Jean Ching-Yi Tien[1,2], Jie Luo[1,2,14], Yu Chang[1,2,14], Yuping Zhang[1,2,14], Yunhui Cheng[1,2,14], , Xiaoju Wang[1,2], Jianzhang Yang[3,4], Rahul Mannan[1,2], Somnath Mahapatra[1,2], Palak Shah[1,2], Xiao-Ming Wang[1,2], Abigail J. Todd[1,2], Sanjana Eyunni[1,2], Caleb Cheng[1], Ryan J. Rebernick[1,2], Lanbo Xiao[1,2], Yi Bao[1,2], James Neiswender[5], Rachel Brough[5], Stephen J. Pettitt[5], Xuhong Cao[1,2], Stephanie J. Miner[1,2], Licheng Zhou[3,4], Yi-Mi Wu[1,2], Estefania Labanca[6], Yuzhuo Wang[7], Abhijit Parolia[1,2,8], Marcin Cieslik[1,2], Dan R. Robinson[1,2], Zhen Wang[3,4], Felix Y. Feng[9,10,11], Jonathan Chou[10,11], Christopher J. Lord[5], Ke Ding[3,*], and Arul M. Chinnaiyan[1,2,8,12,13,15,*]

[1]Michigan Center for Translational Pathology, University of Michigan, Ann Arbor, MI, USA
[2]Department of Pathology, University of Michigan, Ann Arbor, MI, USA
[3]State Key Laboratory of Chemical Biology, Shanghai Institute of Organic Chemistry, Chinese Academy of Sciences, Shanghai 200032, People's Republic of China
[4]International Cooperative Laboratory of Traditional Chinese Medicine Modernization and Innovative Drug Discovery of Chinese Ministry of Education (MOE), Guangzhou City Key Laboratory of Precision Chemical Drug Development, College of Pharmacy, Jinan University, Guangzhou 511400, People's Republic of China
[5]The CRUK Gene Function Laboratory and Breast Cancer Now Toby Robins Research Centre, The Institute of Cancer Research, London, SW3 6JB, UK
[6]Department of Genitourinary Medical Oncology and David H. Koch Center for Applied Research of Genitourinary Cancer, University of Texas MD Anderson Cancer Center, Houston, TX, USA
[7]Vancouver Prostate Centre, Vancouver General Hospital and Department of Urologic Sciences, University of British Columbia, Vancouver, British Columbia, V6H 3Z6, Canada
[8]Rogel Cancer Center, University of Michigan, Ann Arbor, MI, USA
[9]Departments of Radiation Oncology and Urology, University of California, San Francisco, CA, USA
[10]Helen Diller Family Comprehensive Cancer Center, University of California, San Francisco, CA, USA
[11]Division of Hematology/Oncology, Department of Medicine, University of California, San Francisco, CA, USA
[12]Department of Urology, University of Michigan, Ann Arbor, MI, USA
[13]Howard Hughes Medical Institute, University of Michigan, Ann Arbor, MI, USA
[14]These authors contributed equally to this work.
[15]Lead contact

*Correspondence: arul@med.umich.edu (A.M.C.) and dingk@sioc.ac.cn (K.D.)

Figure S1

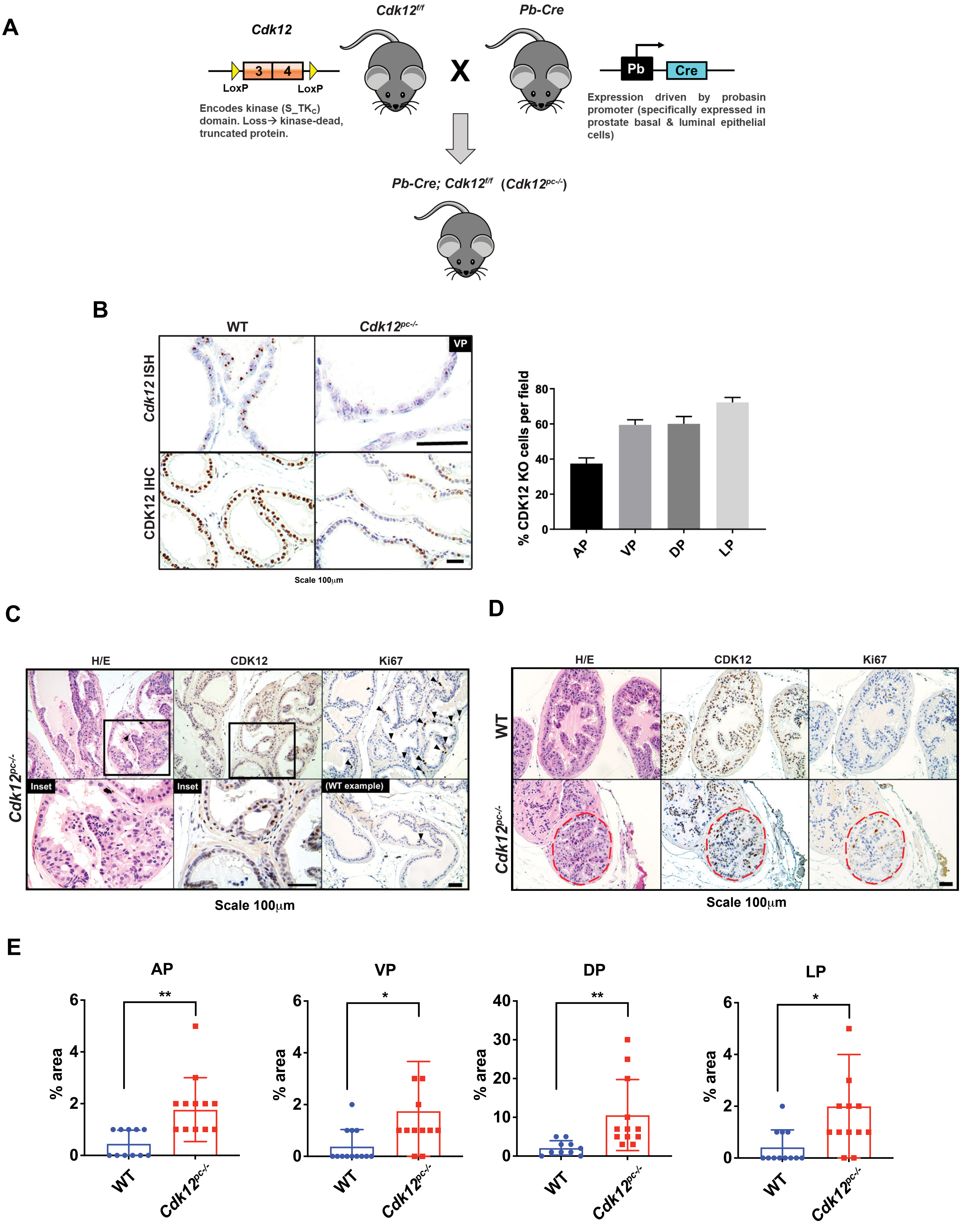

A
Cdk12
3
4
LoxP
LoxP
Encodes kinase (S_TKc) domain. Loss→ kinase-dead, truncated protein.
Cdk12f/f
X
Pb-Cre
Pb
Cre
Expression driven by probasin promoter (specifically expressed in prostate basal & luminal epithelial cells)
Pb-Cre; Cdk12f/f (Cdk12pc-/-)
B
WT
Cdk12pc-/-
VP
Cdk12 ISH
CDK12 IHC
Scale 100μm
% CDK12 KO cells per field
0
20
40
60
80
AP
VP
DP
LP
C
H/E
CDK12
Ki67
Cdk12pc-/-
Inset
Inset
(WT example)
Scale 100μm
D
H/E
CDK12
Ki67
WT
Cdk12pc-/-
Scale 100μm
E
AP
VP
DP
LP
% area
**
*
**
*
0
2
4
6
0
10
20
30
40
WT
Cdk12pc-/-

Figure S1: *Cdk12* is partially ablated in prostate epithelium by *Probasin*-driven Cre recombinase. Related to Figure 1.
(A) Prostate epithelial *Cdk12* ablation scheme.
(B) CDK12 immunohistochemistry (IHC) and *Cdk12 in situ* hybridization (ISH) in 8-week-old WT and $Cdk12^{pc-/-}$ mice. Percent epithelial cells immunonegative for CDK12 (*Cdk12* KO cells) in prostate lobes of $Cdk12^{pc-/-}$ mice: anterior prostate (AP), ventral prostate (VP), dorsal prostate (DP), lateral prostate (LP). (n= 2-3 prostate cross sections from 6 mice).
(C) Hyperplasia with lost nuclear polarity and isonucleosis in prostate epithelium of 30-week-old mixed background $Cdk12^{pc-/-}$ mice. Note concentrated Ki67 staining in histologically abnormal regions. These regions are absent in wild-type (WT) controls.
(D) Larger pre-cancerous lesions (indicated by dashed line) in prostate epithelia of 52-week-old mixed background $Cdk12^{pc-/-}$ mice.
(E) Percent cross sectional area occupied by pre-cancerous lesions in prostate lobes of 52-week-old $Cdk12^{pc-/-}$ mice. Anterior prostate (AP), ventral prostate (VP), dorsal prostate (DP), lateral prostate (LP). (n= 2-3 prostate cross sections from each of 6-7 mice). Data are represented as mean ± SD. Statistical analysis with t-test. *$p<0.05$, **$p<0.01$.

Figure S2

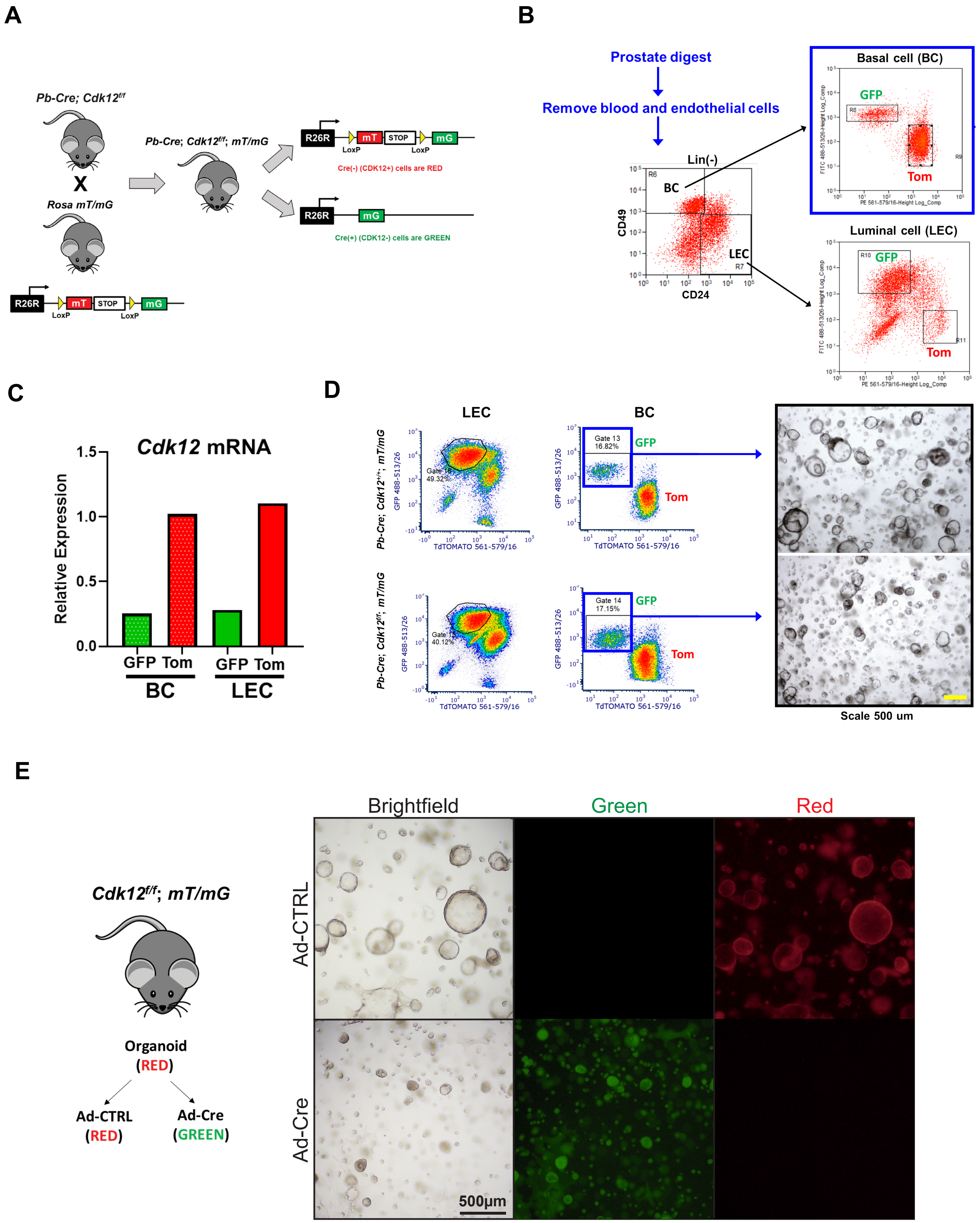
A
Pb-Cre; Cdk12f/f
X
Rosa mT/mG
Pb-Cre; Cdk12f/f; mT/mG
R26R
mT
STOP
mG
LoxP
LoxP
Cre(-) (CDK12+) cells are RED
Cre(+) (CDK12-) cells are GREEN
B
Prostate digest
Remove blood and endothelial cells
Lin(-)
BC
LEC
CD49
CD24
Basal cell (BC)
GFP
Tom
Luminal cell (LEC)
GFP
Tom
C
Cdk12 mRNA
Relative Expression
1.5
1.0
0.5
0.0
GFP
Tom
GFP
Tom
BC
LEC
D
LEC
BC
Pb-Cre; Cdk12+/+; mT/mG
Pb-Cre; Cdk12f/f; mT/mG
Gate 13
16.82%
Gate 14
17.15%
GFP
Tom
GFP 488-513/26
TdTOMATO 561-579/16
Scale 500 um
E
Cdk12f/f; mT/mG
Organoid
(RED)
Ad-CTRL
(RED)
Ad-Cre
(GREEN)
Brightfield
Green
Red
Ad-CTRL
Ad-Cre
500μm

Figure S2: Application of *mT/mG* model to isolate cells with active Cre recombinase and *Cdk12* ablation; demonstration of abnormal morphology in organoids generated from *Cdk12*-null cells. Related to Figure 2.
(A) Generation of a *Pb-Cre*;*Cdk12*$^{f/f}$;*mT/mG* prostate mouse model to identify prostate epithelial cells with active Cre recombinase.
(B) Basal cell isolation from *Pb-Cre*;*Cdk12*$^{f/f}$;*mT/mG* prostate (52-week time point).
(C) *Cdk12* mRNA expression in 52-week *Pb-Cre*;*Cdk12*$^{f/f}$;*mT/mG* prostate epithelial cells. BC, basal cells; LEC, luminal epithelial cells.
(D) Confirmatory experiment demonstrating that enhanced GFP(+)/ Cre-expressing cells from *Cdk12*$^{+/+}$ mouse prostate do not have abnormal organoid phenotype.
(E) Acute *Cdk12* ablation achieved through *in vitro* adenoviral Cre to *Cdk12*$^{f/f}$;*mT/mG* organoids. Ad-CTRL indicates control adenovirus. Ad-Cre indicates Cre-expressing adenovirus. Ablation of *Cdk12* gene is coupled with red (Tom) to green (GFP) color change. Images show morphology of organoids with WT *Cdk12* (Ad-CRTL) and *Cdk12* ablation (Ad-Cre).

Figure S3

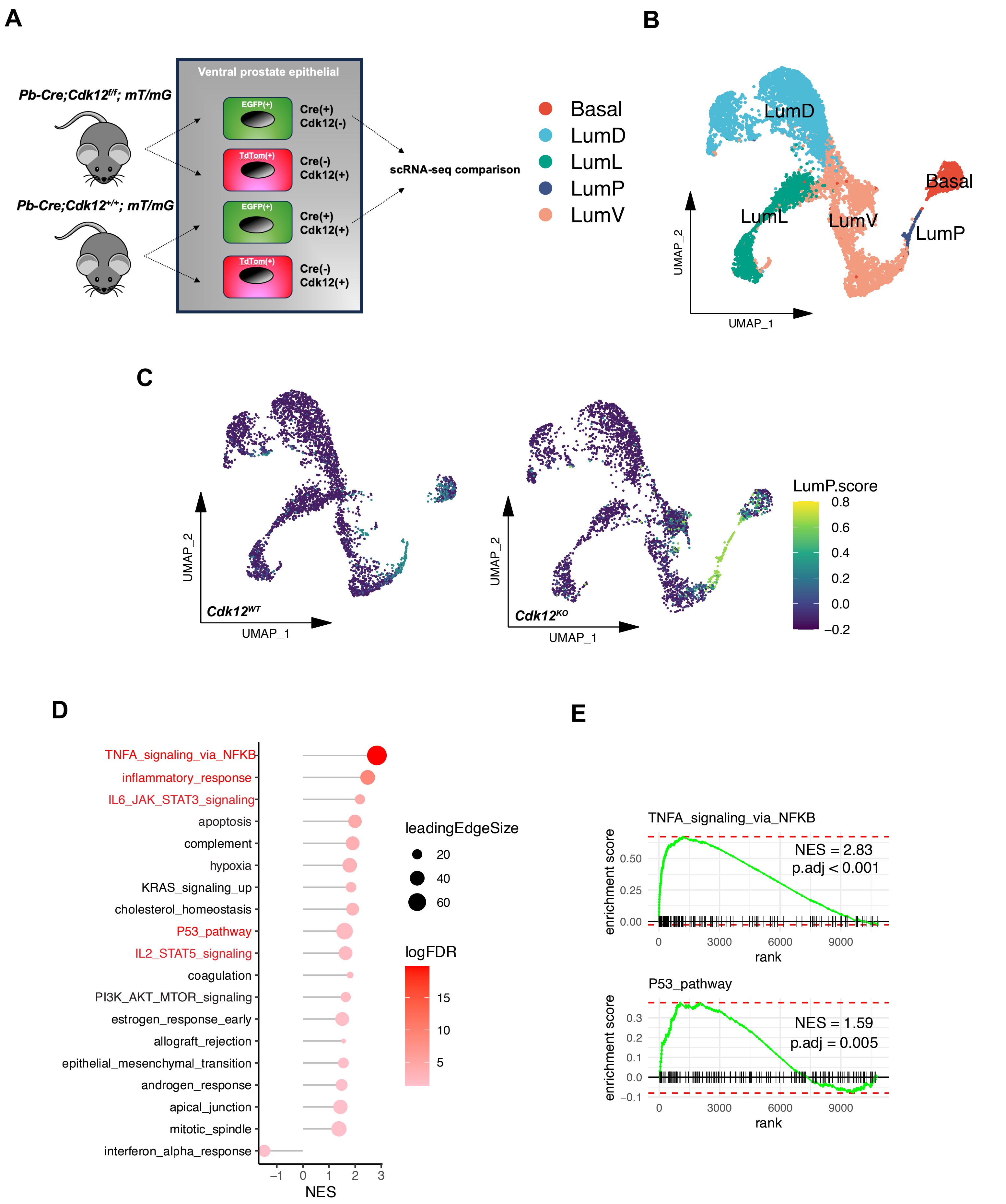
A
Pb-Cre;Cdk12f/f; mT/mG
Pb-Cre;Cdk12+/+; mT/mG
Ventral prostate epithelial
EGFP(+)
Cre(+)
Cdk12(-)
TdTom(+)
Cre(-)
Cdk12(+)
EGFP(+)
Cre(+)
Cdk12(+)
TdTom(+)
Cre(-)
Cdk12(+)
scRNA-seq comparison
B
Basal
LumD
LumL
LumP
LumV
UMAP_2
UMAP_1
C
Cdk12WT
Cdk12KO
LumP.score
0.8
0.6
0.4
0.2
0.0
−0.2
D
TNFA_signaling_via_NFKB
inflammatory_response
IL6_JAK_STAT3_signaling
apoptosis
complement
hypoxia
KRAS_signaling_up
cholesterol_homeostasis
P53_pathway
IL2_STAT5_signaling
coagulation
PI3K_AKT_MTOR_signaling
estrogen_response_early
allograft_rejection
epithelial_mesenchymal_transition
androgen_response
apical_junction
mitotic_spindle
interferon_alpha_response
NES
leadingEdgeSize
20
40
60
logFDR
15
10
5
E
TNFA_signaling_via_NFKB
NES = 2.83
p.adj < 0.001
enrichment score
rank
P53_pathway
NES = 1.59
p.adj = 0.005

Figure S3: *Cdk12* null mice exhibit upregulation of specific prostate cancer-associated pathways and p53 target genes. Related to Figure 3.

(A) Scheme for scRNA-seq analysis of prostates from *mT/mG* mice with wild-type (WT) Cdk12 (*Cdk12*$^{+/+}$) or prostate epithelial-specific *Cdk12* ablation (*Cdk12*$^{f/f}$) driven by *Probasin*-Cre (*Pb-Cre*) (KO). Cells of mT/mG mice express td-Tomato (TdTom) at baseline. The TdTom sequence is excised in cells with active Cre recombinase, enabling expression of enhanced GFP (EGFP). The strategy outlined above allows for comparison of only Cre-expressing cells (i.e., EGFP-expressing cells) from each animal. (n= 3 mice per group)

(B) UMAP plot indicating cell populations from cells described in (A). Cells are annotated using the Crowely et al. dataset as reference[72]. LumD, LumL, and LumV are Luminal cells specific to dorsal, lateral, and ventral prostate, respectively; LumP is the proximal progenitor population.

(C) Enrichment of Lum P population in *Cdk12*-null GFP(+) cells (*Cdk12*^{KO}).

(D) Enrichment of cancer-related pathways in LumV cells of the *Cdk12*^{KO} prostate epithelium.

(E) Enrichment plots of selected pathways enriched in luminal cells from the *Cdk12*^{KO} ventral prostate.

Figure S4

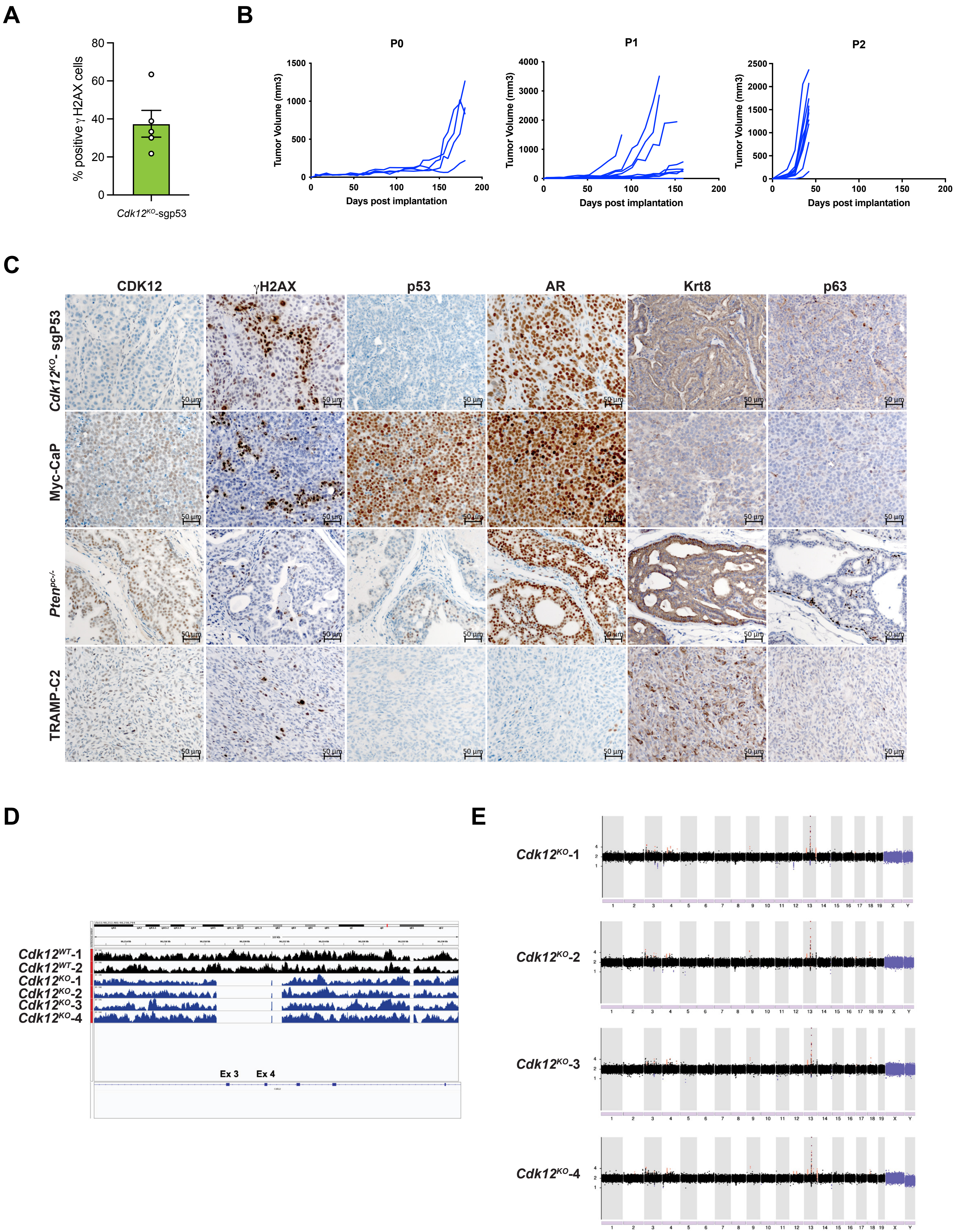
A
B
C
D
E
% positive γH2AX cells
Cdk12KO-sgp53
P0
P1
P2
Tumor Volume (mm3)
Days post implantation
CDK12
γH2AX
p53
AR
Krt8
p63
Cdk12KO- sgP53
Myc-CaP
Ptenpc-/-
TRAMP-C2
50 µm
Cdk12WT-1
Cdk12WT-2
Cdk12KO-1
Cdk12KO-2
Cdk12KO-3
Cdk12KO-4
Ex 3
Ex 4

Figure S4: Clonal *Cdk12*^{KO} organoid lines do not demonstrate focal tandem duplications. Related to Figure 4.
(A) Bar graph indicates percent γH2AX positive cells from average of 5 sections evaluated from each of 3 mice. Data are represented as mean ± SEM.
(B) *Cdk12/Trp53* double KO organoid cells serially passaged as subcutaneous allografts in mice. Each line represents an individual allograft.
(C) Immunohistochemical staining of CDK12, γH2AX, AR, Krt8, and p53 in *Cdk12*^{KO}-sgp53 allografts, Myc-CaP allografts, and TRAMP-C2 allografts, and prostates of the established *Pten*$^{pc-/-}$ prostate cancer mouse model. Scale bar indicates 50µm.
(D) Genomic sequencing of clonal *Cdk12*^{KO} organoid lines (*Cdk12*^{KO}-1, *Cdk12*^{KO} -2, *Cdk12*^{KO}-3, *Cdk12*^{KO}-4) demonstrating ablation of exons 3 and 4. Each plot indicates sequencing of an individual monoclonal organoid line.
(E) Genomic sequencing of *Cdk12*^{KO} organoid lines demonstrating that these do not have evidence of the focal tandem duplication pattern seen in human prostate cancer lacking functional *CDK12*. Each plot indicates sequencing of an individual monoclonal organoid line.

Figure S5

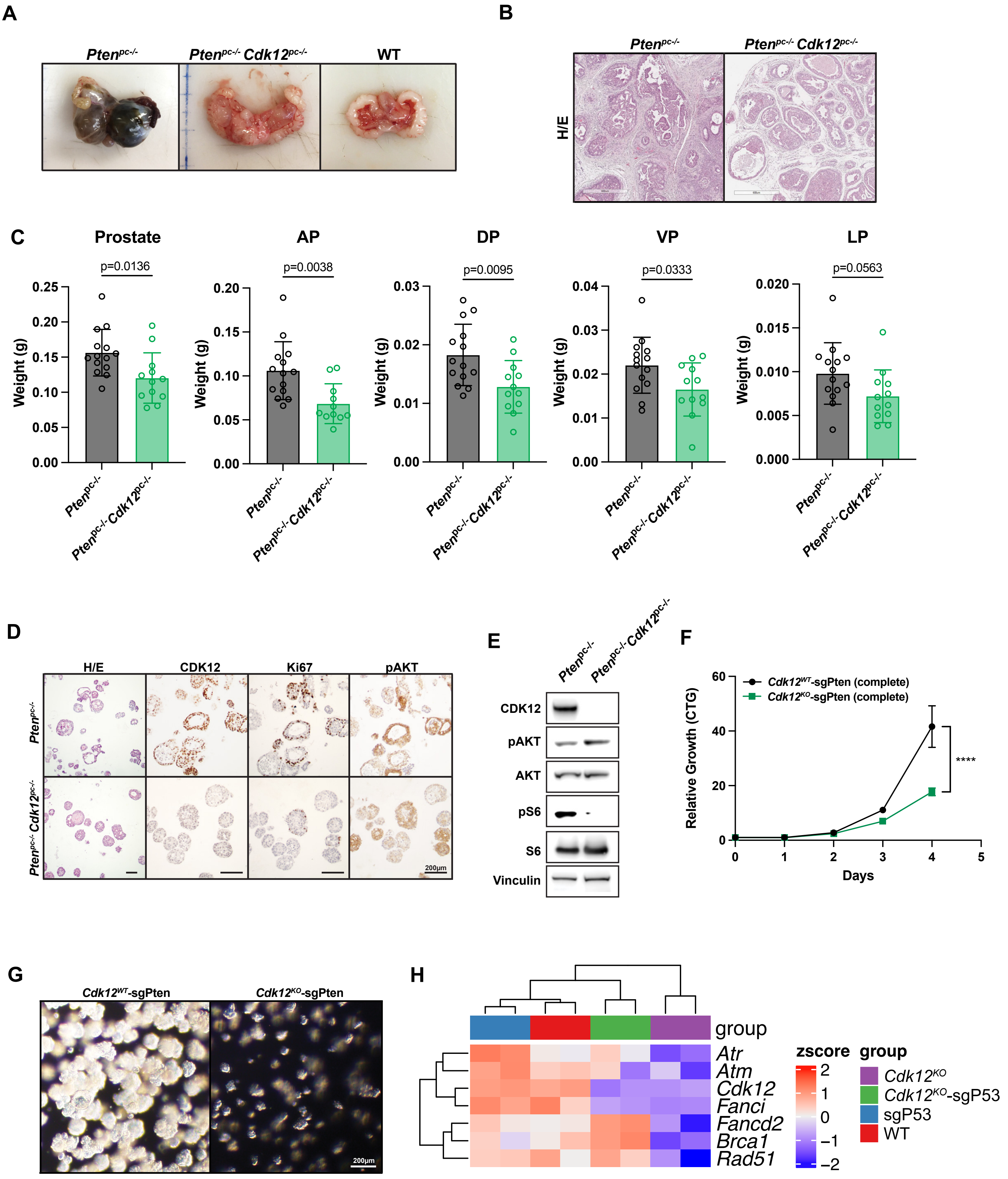
A
Ptenpc-/-
Ptenpc-/- Cdk12pc-/-
WT
B
Ptenpc-/-
Ptenpc-/- Cdk12pc-/-
H/E
C
Prostate
p=0.0136
AP
p=0.0038
DP
p=0.0095
VP
p=0.0333
LP
p=0.0563
Weight (g)
D
H/E
CDK12
Ki67
pAKT
200µm
E
CDK12
pAKT
AKT
pS6
S6
Vinculin
F
Cdk12WT-sgPten (complete)
Cdk12KO-sgPten (complete)
Relative Growth (CTG)
Days
****
G
Cdk12WT-sgPten
Cdk12KO-sgPten
200µm
H
group
zscore
Atr
Atm
Cdk12
Fanci
Fancd2
Brca1
Rad51
Cdk12KO
Cdk12KO-sgP53
sgP53
WT

Figure S5: *Cdk12* ablation impairs tumor progression in the *Pten*-null mouse model of prostate cancer. Related to Figure 4.

(A) Representative images of GU tracts from mice indicated in (B).

(B) H/E-stained sections of *Pten*$^{pc-/-}$ and *Pten*$^{pc-/-}$ *Cdk12*$^{pc-/-}$ prostate. Scale bars indicate 600µm.

(C) Weights of whole prostate and individual lobes of *Pten*$^{pc-/-}$ and *Pten*$^{pc-/-}$ *Cdk12*$^{pc-/-}$ mice at 24 weeks.

(D) Immunohistochemical staining of CDK12, Ki67, and phosphorylated AKT (pAKT) in cross sections of organoids described in (F). Scale bars indicate 200µm.

(E) Protein expression of CDK12, pAKT, and pS6 in *Pten*$^{pc-/-}$ and *Pten*$^{pc-/-}$ *Cdk12*$^{pc-/-}$ organoids with vinculin serving as a loading control.

(F) Cell proliferation of basal cell-derived *Cdk12*^{WT} and *Cdk12*^{KO} organoids subjected to CRISPR-mediated *Pten* ablation (sgPten) as measured by CTG assay. (n= 4 samples per group)

(G) Phase contrast images of organoids described in (F).

Data are represented as mean ± SEM. Log-rank (Mantel-Cox) test was used to detect significance in (A). One-way ANOVA test was used to detect significance in (B). Unpaired t test was used for tumor weight in (E). Two-way ANOVA test was used for (F) and (I) ****p<0.0001.

(H) Expression of DNA damage response genes in *Cdk12*^{KO}, sgp53, and *Cdk12*^{KO}-sgp53 organoids by RNA-seq.

Figure S6

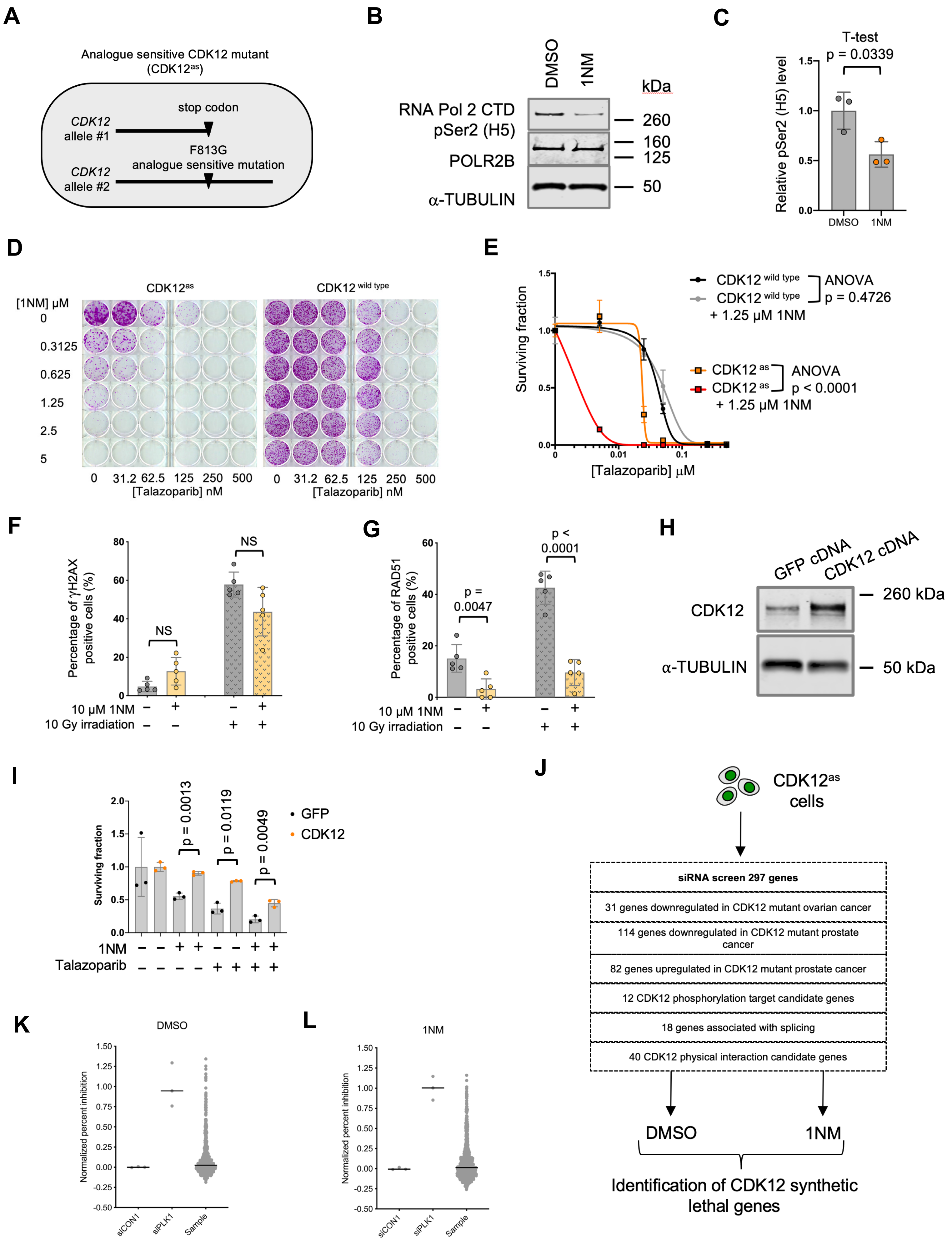
A
Analogue sensitive CDK12 mutant (CDK12as)
CDK12 allele #1
stop codon
CDK12 allele #2
F813G analogue sensitive mutation
B
DMSO
1NM
kDa
RNA Pol 2 CTD pSer2 (H5)
POLR2B
α-TUBULIN
260
160
125
50
C
T-test
p = 0.0339
Relative pSer2 (H5) level
DMSO
1NM
D
CDK12as
CDK12 wild type
[1NM] μM
0
0.3125
0.625
1.25
2.5
5
0 31.2 62.5 125 250 500
[Talazoparib] nM
E
Surviving fraction
[Talazoparib] μM
CDK12 wild type
CDK12 wild type + 1.25 μM 1NM
ANOVA p = 0.4726
CDK12 as
CDK12 as + 1.25 μM 1NM
ANOVA p < 0.0001
F
Percentage of γH2AX positive cells (%)
NS
NS
10 μM 1NM
10 Gy irradiation
G
Percentage of RAD51 positive cells (%)
p = 0.0047
p < 0.0001
10 μM 1NM
10 Gy irradiation
H
GFP cDNA
CDK12 cDNA
CDK12
α-TUBULIN
260 kDa
50 kDa
I
Surviving fraction
p = 0.0013
p = 0.0119
p = 0.0049
GFP
CDK12
1NM
Talazoparib
J
CDK12as cells
siRNA screen 297 genes
31 genes downregulated in CDK12 mutant ovarian cancer
114 genes downregulated in CDK12 mutant prostate cancer
82 genes upregulated in CDK12 mutant prostate cancer
12 CDK12 phosphorylation target candidate genes
18 genes associated with splicing
40 CDK12 physical interaction candidate genes
DMSO
1NM
Identification of CDK12 synthetic lethal genes
K
DMSO
Normalized percent inhibition
siCON1
siPLK1
Sample
L
1NM
Normalized percent inhibition
siCON1
siPLK1
Sample

Figure S6: Validation of CDK12^{as} cells and overview of siRNA screen. Related to Figure 6.
(**A**) Diagram indicating generation of an analog sensitive CDK12 mutant HeLa cell line (CDK12^{as}) (in lab of Arno Greenleaf) by two rounds of CRISPR mutagenesis; the first introduced a stop mutation into one *CDK12* allele and the second introduced a homologous recombination template coding for an p.F813G amino acid change in the DFG sequence of the CDK12 kinase domain. This alteration causes the kinase domain to take a wider configuration and to be inhibited by the adenine analog 1-NM-PP1 (1NM).
(B) Western blot indicating the effect of CDK12 inhibition on phosphorylation of the CTD of RNA Pol-II in CDK12^{as} cells exposed to 1NM.
(C) Quantification of relative protein level shown in (B) from three independent experiments. Error bars represent standard error of the mean (SEM). p value calculated by t-test.
(D) Colony formation assay (CFA) images from CDK12^{as} or CDK12 wild-type cells (CDK12 $^{wild\text{-}type}$) exposed to 1NM and/or the PARP inhibitor talazoparib.
(E) Quantification of CFA data from replica experiments. Dots represent the mean and error bars represent SEM. p value calculated by two-way ANOVA.
(F, G) Quantification of irradiation-induced nuclear gH2AX and RAD51 nuclear foci in CDK12^{as} cells exposed to 1NM. Dots represent the percentage of cells with 5 or more detectable nuclear foci. Columns indicate the median score from five experiments. Error bars represent SEM. p values calculated by t-test.
(H) Western blot showing overexpression of a wild-type CDK12 cDNA construct in CDK12^{as} cells.
(I) Wild-type CDK12 expression reduces 1NM-induced PARP inhibitor sensitivity. CellTiter-Glo results from 1 week of growth normalized to DMSO treated controls. Columns indicate the median of 6 biological replicate samples, indicated by individual dots. p value calculated by t-test.
(J) Schematic of siRNA screen. CDK12^{as} cells were transfected in a 96 well plate format with a custom siRNA library targeting genes annotated as shown. Positive (siPLK1) and negative controls (siCON1, Dharmacon) were also included in each plate. After 24 hours, media was replaced with new media drug containing 1NM (0.3 mM) or the drug vehicle (DMSO) and cells were continuously cultured for six further days, at which point cell viability was estimated by the use of CellTiter-glo reagent.
(K, L) Quality control data from siRNA screen. Normalized percent inhibition (NPI) data is shown for non-targeting control siRNA (siCON1, normalized at NPI = 1 from three replica screens), siRNA targeting PLK1 (normalized at NPI = 1 from three replica screens), and siRNA designed to target each of the genes included in the screen. Data from DMSO-exposed (K) and 1NM-exposed (L) arms of the screen are shown, indicating a large dynamic range between siCON1 and siPLK1.

Figure S7

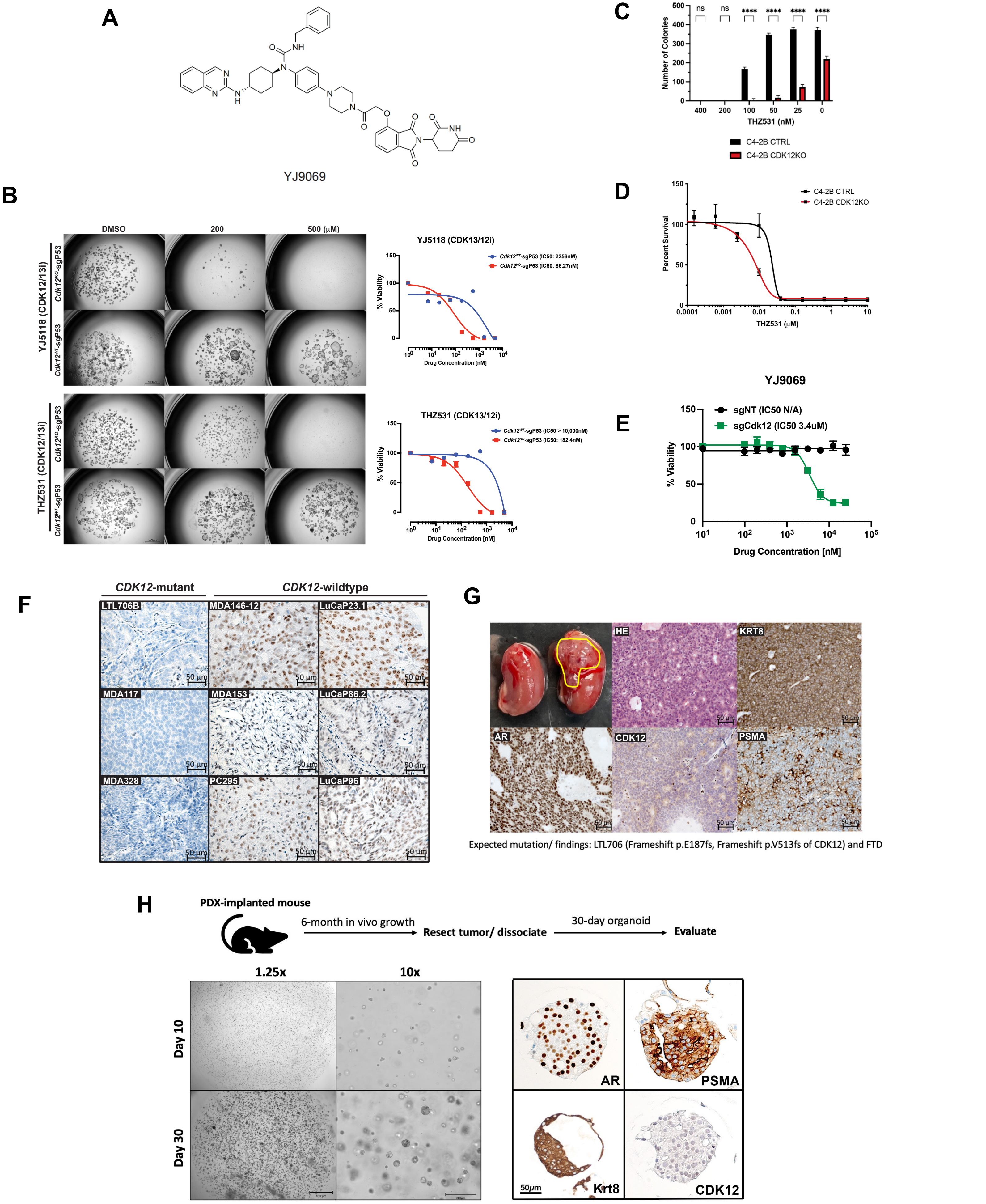
A
YJ9069
B
DMSO
200
500 (μM)
YJ5118 (CDK12/13i)
Cdk12KO-sgP53
Cdk12WT-sgP53
THZ531 (CDK12/13i)
YJ5118 (CDK13/12i)
Cdk12WT-sgP53 (IC50: 2256nM)
Cdk12KO-sgP53 (IC50: 86.27nM)
% Viability
Drug Concentration [nM]
THZ531 (CDK13/12i)
Cdk12WT-sgP53 (IC50 > 10,000nM)
Cdk12KO-sgP53 (IC50: 182.4nM)
C
ns
****
Number of Colonies
THZ531 (nM)
C4-2B CTRL
C4-2B CDK12KO
D
Percent Survival
THZ531 (μM)
E
YJ9069
sgNT (IC50 N/A)
sgCdk12 (IC50 3.4uM)
Drug Concentration [nM]
F
CDK12-mutant
CDK12-wildtype
LTL706B
MDA146-12
LuCaP23.1
MDA117
MDA153
LuCaP86.2
MDA328
PC295
LuCaP96
50 μm
G
HE
KRT8
AR
CDK12
PSMA
Expected mutation/ findings: LTL706 (Frameshift p.E187fs, Frameshift p.V513fs of CDK12) and FTD
H
PDX-implanted mouse
6-month in vivo growth
Resect tumor/ dissociate
30-day organoid
Evaluate
1.25x
10x
Day 10
Day 30
AR
PSMA
Krt8
CDK12
50μm

Figure S7: CDK13/12 inhibitor treatment in organoids and PDX model with *CDK12* loss of function. Related to Figures 6-7.
(A) Chemical structure of YJ9069.
(B) *Cdk12*^{WT}-sgp53 and *Cdk12*^{KO}-sgp53 organoids treated with CDK13/12 inhibitors (YJ5118, THZ531). Bright field images show organoids subjected to each agent at concentrations of 200 and 500 µM (and DMSO vehicle control). Line graphs show IC_{50} curves for both organoid types treated with each agent. (n= 3 samples per group, 2 individual experiments). Scale indicates 1000µm.
(C) C4-2B *CDK12*KO and C4-2B CTRL cells: Colony formation assay performed with increasing THZ531 concentrations.
(D) C4-2B *CDK12*KO and C4-2B CTRL cells: Percent viability at increasing THZ531 concentrations.
(E) IC_{50} values of *Cdk12* knockout (sgCdk12) and control (sgNT) Myc-CaP cells treated with CDK12/13 degrader YJ9069.
(F) CDK12 IHC of the indicated prostate PDX lines. Scale indicates 50µm.
(G) PDX line LTL706B (biallelic frameshift mutations in *CDK12* gene) in mouse kidney. Tumor tissue stained for AR, KRT8, PSMA, and CDK12. Scale indicates 50µm.
(H) Scheme for organoid generation from LTL706B tumors. Organoids derived from LTL706B tumors in bright field (left panel, scale 1000µm; right panel, scale 200µm) and embedded/cross-section stained for AR, KRT8, PSMA, and CDK12 (scale 50µm).
